# Immune regulation by low doses of the DNA methyltransferase inhibitor 5-azacitidine in common human epithelial cancers

**DOI:** 10.18632/oncotarget.1782

**Published:** 2014-02-16

**Authors:** Huili Li, Katherine B. Chiappinelli, Angela A. Guzzetta, Hariharan Easwaran, Ray-Whay Chiu Yen, Rajita Vatapalli, Michael J. Topper, Jianjun Luo, Roisin M. Connolly, Nilofer S. Azad, Vered Stearns, Drew M. Pardoll, Nancy Davidson, Peter A. Jones, Dennis J. Slamon, Stephen B. Baylin, Cynthia A. Zahnow, Nita Ahuja

**Affiliations:** ^1^ Department of Oncology, The Sidney Kimmel Comprehensive Cancer Center at Johns Hopkins, Baltimore, MD, USA; ^2^ Breast Cancer Program, The Sidney Kimmel Comprehensive Cancer Center at Johns Hopkins, Baltimore, MD, USA; ^3^ Department of Medicine, University of Pittsburgh Cancer Institute and UPMC CancerCenter, Pittsburgh, PA; ^4^ Departments of Urology and Biochemistry and Molecular Biology, USC Norris Comprehensive Cancer Center, Keck School of Medicine, University of Southern California, Los Angeles, CA; ^5^ The Jonsson Comprehensive Cancer Center, University of California-Los Angeles; ^6^ Department of Surgery, School of Medicine, Johns Hopkins University, Baltimore, MD, USA

**Keywords:** Epigenetics, immune, cancers, DNA methyltransferase inhibitor, interferon, methylation, antigen processing

## Abstract

Epigenetic therapy is emerging as a potential therapy for solid tumors. To investigate its mechanism of action, we performed integrative expression and methylation analysis of 63 cancer cell lines (breast, colorectal, and ovarian) after treatment with the DNA methyltransferase inhibitor 5-azacitidine (AZA). Gene Set Enrichment Analysis demonstrated significant enrichment for immunomodulatory pathways in all three cancers (14.4-31.3%) including interferon signaling, antigen processing and presentation, and cytokines/chemokines. Strong upregulation of cancer testis antigens was also observed. An AZA IMmune gene set (AIMs) derived from the union of these immunomodulatory pathway genes classified primary tumors from all three types into “high” and “low” AIM gene expression subsets in tumor expression data from both TCGA and GEO. Samples from selected patient biopsies showed upregulation of AIM genes after treatment with epigenetic therapy. These results point to a broad immune stimulatory role for DNA demethylating drugs in multiple cancers.

## INTRODUCTION

Cancers are now recognized as being driven by widespread changes in the epigenome including changes in DNA methylation and chromatin packaging [1]. Changes in DNA methylation include global loss of methylation and focal gain of methylation at promoter regions of tumor suppressor genes leading to transcriptional silencing [1]. DNA methylation, a covalent modification of DNA, is mediated by a family of DNA methyltransferases (DNMTs). In recent years, inhibitors of DNMTs (DNMTis) have emerged as therapeutic targets for treatment of myeloid malignancies as well as cutaneous T cell lymphoma. FDA approval was given to the DNMT inhibitor 5-azacitidine (AZA) for treatment of myelodysplastic syndrome in 2004 [2]. Several groups, including ours, have focused on the therapeutic potential of DNMT inhibitors in the treatment of solid tumors with exciting early possibilities seen in non-small cell lung cancer (NSCLC) [3] and reversal of chemotherapy resistance in ovarian cancers [4]. Recently, our group has also seen exciting robust clinical responses in a small number of patients with NSCLC who received therapy to break immune tolerance after epigenetic therapy with AZA, along with an HDAC inhibitor (HDACi), entinostat [5].

Much of our above clinical trial work was driven by our pre-clinical studies that showed how low doses of DNMTis may avoid off-target effects, mimic doses seen by patients' tumor cells, and reprogram and inhibit tumor cells, including cancer stem-like cells [6]. We have now investigated, first using this pre clinical paradigm, the global response of 63 cultured cell lines to transient, low-dose AZA in three common human cancers (breast, colorectal and ovarian) by studying the expression and methylation changes at multiple time points. We demonstrate that AZA can upregulate a defined set of immunomodulatory pathways (based on Gene Set Enrichment Analysis (GSEA)) in all three cancer types and we derive a gene panel reflecting this which we term AZA IMmune genes (AIMs). We show how this panel divides primary human cancers in all three cancer types, and other cancer such as NSCLC and melanoma, into a “low” and “high” AIM signature. Importantly, increased expression of AIM genes could also be seen, in subsets of patients treated with AZA in breast and colorectal clinical trials, in a comparison of pre- and post- treatment biopsy samples, suggesting that epigenetic treatment causes enrichment, *in vivo*, of immunomodulatory genes. Our data shows that the AIM gene panel stratifies patients with common human cancers into an immune low and immune enriched group and suggests that patients with low expression of AIM genes would benefit from epigenetic therapy when combined with immunotherapy.

## RESULTS

**Figure 1:**
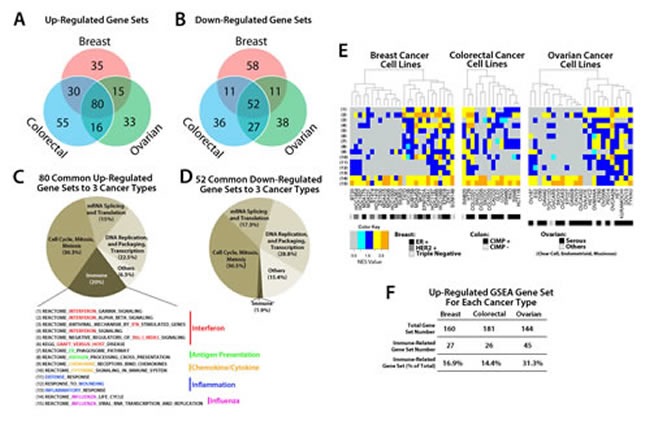
GSEA analysis of transcripts regulated by AZA in breast, colorectal, and ovarian cancer cell lines reveals pathways common to all three cancer types Venn Diagram showing the number of GSEA gene sets A) upregulated (NES > 2.15, FDR < 0.25) and B) downregulated (NES <−2.15, FDR < 0.25) by AZA in breast, colorectal, and ovarian cell lines. Agilent array data were normalized and analyzed by GSEA. Pie charts of gene sets common to all three cancer types that were C) upregulated and D) downregulated show the different categories of the common GSEA pathways. The “Immune” sector is broken down further into specific pathways characterized as part of the interferon response, antigen presentation, cytokines/chemokines, inflammation, and influenza virus. E) Heat maps showing the NES value from GSEA for each cell line (x axis) and each of the 15 immune pathways (y-axis) shown in C. The colored rectangle corresponding to NES is graded from gray (low) to orange (high). Subtypes for each cancer type are coded by the black, grey, and white boxes shown below the figure. F) Summary of GSEA gene sets upregulated by AZA in each cancer type and the percent that were immune-related.

We explored further our understanding of the global pathway changes after treatment with low doses of the DNMTi AZA in cell lines from multiple common human cancers. A total of 63 cancer cell lines (26 breast, 14 colorectal, and 23 ovarian) were treated with low-dose (500 nM) AZA for three days. DNA and RNA were isolated at multiple time points following initial drug application and analyzed for genome-wide changes in DNA methylation (Illumina Infinium 450K) and gene expression (Agilent 44K Expression Array). We used these genomics data to identify the most enriched pathway alterations as analyzed by GSEA [7] (Fig. 1, Fig. S1) focusing upon the ~top 30% of all upregulated and downregulated gene sets. GSEA analyses of AZA inducible genes identified 80 upregulated gene sets and 52 downregulated gene sets that were common between the three cancer types (Fig. 1a,b; Fig. S1). These gene sets could be broadly divided into four categories including cell cycle control (cell cycle, mitosis, meiosis), DNA replication (DNA replication and packaging, transcription), mRNA splicing and translation, and immune response (Fig. 1c,d; Table S1a,b). The majority of the immune gene sets showed upregulation by AZA (15/16 gene sets or 93.7%) except for the “systemic lupus erythematosus” gene set, which also showed downregulation (Table S1a,b). We thus focused the remainder of our analysis on those immune gene sets that only showed upregulation in response to AZA.

The abovementioned 15 upregulated immune gene sets (Fig. 1c) were classified as interferon signaling, antigen presentation, chemokine and cytokine signaling, inflammation, and influenza (Fig. 1c, Table 1). These immune pathways were activated in almost every cell line in response to AZA and did not cluster with a specific subtype of cancer (for example, receptor status in breast cancers, CpG Island Hypermethylator Phenotype (CIMP) [8], or histologic subtype) (Fig. 1e). Overall immune pathway upregulation was highest in ovarian cancers (31.3%) followed by breast (16.9%) and colorectal (14.4%) (Fig. 1f). We compared these 80 upregulated gene sets from our three cancer types to 14 lung cancer cell lines that had been treated with the same AZA dosing schedule [5]. Interestingly, 76/80 (95%) of the gene sets common to breast, colorectal, and ovarian cell lines (Fig. 1a) were also upregulated in the lung cancer cell lines. In addition, 23.3% of significantly upregulated pathways in the lung cancer cell lines were also immune related. This suggested to us that AZA drives common signaling pathways in many solid tumor types and immunomodulatory pathways are a significant fraction of these AZA upregulated pathways.

**Table 1:** AZA Immune Genes (AIMs)

Gene Set Categories	Common Genes in 3 Types of Cancer	Common Genes in Any 2 Types	Unique Genes		
			Breast	Colorectal	Ovarian
**Interferon (42.3%)**	B2M; CD44; GBP1; HLA-B; HLA-C; ICAM1; IRF7; IRF9; MT2A; OAS1; OAS2; OAS3; OASL; STAT1; EGR1; IFI27; IFI6; IFIT1; IFIT2; IFIT3; IFITM1; ISG15; ISG20; MX1; PSMB8; USP18; XAF1; DDX58; HERC5; UBA7; IFIH1; TNFAIP3	HLA-DRB1; EIF4E; EIF4G1; NUP35; UBE2L6; GBP2; HLA-A; HLA-DPB1; HLA-F; IFITM2; IFITM3; MX2; UBE2E1; FAS; FASLG; HLA-DMA; HLA-E; GBP5; IFNGR1; IRF6; VCAM1; IL1A; IL1B; IL6	IRF8; JAK2; EIF2AK2; TPR; NLRX1; HLA-DMB	CAMK2B; HLA-DRB3; PTAFR; PTPN1; EIF4A2; KPNA2; KPNA3; NUP107; NUP155; NUP205; NUP37; NUP43; NUP85; NUP93; SEH1L; UBE2N; GZMB; PRF1	GBP4; HLA-DPA1; HLA-G; IFNG; PTPN6; IFI35; RNASEL; STAT2
**Cytokine/Chemokine (43.7%)**	CCL2; CCL20; CCL5; CXCL1; CXCL11; CXCL2; CXCL3; CXCL6; CXCR4; IL8; B2M; CD44; CSF2; DDX58; EGR1; GBP1; HERC5; HLA-B; HLA-C; ICAM1; IFI27; IFI6; IFIT1; IFIT2; IFIT3; IFITM1; IL1R2; IRF7; IRF9; ISG15; ISG20; LCK; MT2A; MX1; OAS1; OAS2; OAS3; OASL; PSMB8; STAT1; UBA7; USP18; XAF1	CCL4; PPBP; EIF4E; EIF4G1; HLA-DRB1; LYN; NUP35; UBE2L6; CASP1; GBP2; HLA-A; HLA-DPB1; HLA-F; IFITM2; IFITM3; IL18; IL6R; IL7R; MX2; NFKB2; UBE2E1; CCL28; CCL3; CCL3L3; CXCR7; GBP5; IFNGR1; IL1A; IL1B; IL6; IRF6; NOD2; STAT5A; VCAM1	CCR9; CXCL12; CXCL9; EIF2AK2; IL6ST; IRF8; JAK2; PIK3R2; TPR	CCR7; CXCL10; CXCL16; CXCR3; PF4; CAMK2B; CDK1; CSF2RB; EIF4A2; HLA-DRB3; HRAS; IL1R1; IL1RN; IL2RA; IL2RG; IRAK1; KPNA2; KPNA3; MAP2K4; NRAS; NUP107; NUP155; NUP205; NUP37; NUP43; NUP85; NUP93; PELI3; PRL; PTAFR; PTPN1; RBX1; SEH1L; SH2B1; SHC1; UBE2N	CCRL1; CXCL5; CXCR6; XCL1; XCL2; CSF2RA; CSH1; GBP4; GH1; HLA-DPA1; HLA-G; IFI35; IFNG; IL2RB; MAP3K8; PELI1; PELI2; PTPN6; RNASEL; STAT2; VAV1
**Ag Presentation (35.5%)**	B2M; HLA-B; HLA-C; PSMB8; PSMB9; TAP1; CTSS; NCF2	PSMA3; CALR; HLA-A; HLA-F; PSME2; ITGAV	PSMC6; MRC2	PSMA6; PSMB10; PSMB3; PSMB6; PSMD1; PSMD10; SEC61B; SEC61G; ITGB5	HLA-G; CD36
**Inflammation (44.2%)**	ALOX5AP; ANKRD1; AOX1; CCL20; CCL26; CCL5; CXCL1; CXCL11; CXCL2; CXCL6; CXCR4; EREG; FOS; HCP5; HLA-B; IL32; IL8; KCNN4; KLRC2; LSP1; LY96; LYST; MX1; NCF2; PAGE1; RSAD2; S100A8; ADM; C4BPB; CTGF; KLK8; MDK; PLAT; SERPINE1; SPRR3; TFPI; THBD	ADORA2B; ANXA1; AOC3; CAMP; CCL4; NLRP3; WAS; APOBEC3G; BNIP3; CD19; CEBPB; CEBPG; DEFB1; HP; INHBB; KLRC4; LY75; MX2; NMI; SCG2; TCIRG1; TLR3; TPST1; VWF; CCL3; CCL3L3; FOSL1; IL1A; INHBA; NOD2; PLA2G7; PTX3; S100A7; S100A9; TYROBP; DCBLD2; GP9; PROS1	ADORA2A; BCL2; CCR9; CD81; CRP; CXCL9; DEFB103A; LBP; NCF1; ORM1; ORM2; TGFB2	AFAP1L2; AIF1; APOBEC3F; CADM1; CCR7; CD83; CXCL10; CYSLTR1; GAGE1; IL17RB; KLRC3; LGALS3BP; LYZ; MGLL; MICB; NFATC4; NOS2; OR2H2; PRF1; PSG8; PTAFR; PYDC1; S100A12; TFF3; UMOD; F2; F2R; F5; F7; MIA3; PF4; SOD1	APOL3; BNIP3L; C2; CD1D; CD40; CFP; CHST2; COLEC12; DCDC2; DMBT1; ELF3; GPR68; HLA-G; IL29; KRT1; MST1R; NOX4; SP140; STAB1; TNFAIP6; TNIP1; CD36; F12; HOXB13; LYVE1; PROC
**Influenza (17.7)**	HSP90AA1; RPL26	NUP35; RPL38; XPO1; CALR; RPS27; RPS8	TPR	GTF2F2; NUP107; NUP155; NUP205; NUP37; NUP43; NUP85; NUP93; POLR2K; POLR2L; RPL11; RPL12; RPL14; RPL15; RPL37A; RPL4; RPL41; RPLP1; RPS11; RPS14; RPS18; RPS23; RPS28; RPS4Y1; RPS6; SEH1L	RPS12
**Cancer Testis Antigens (31.4%)**	ATAD2; CABYR; CSAG1 CT45A1; CT4SAS; CT47A11; CTAG1A; CTAG2; CTCFL; DDX43; DSCRB; FAM133A; FMR1NB; GAGE7; HORMAD1; IL13RA2; MAEL; MAGEA10; MAGEA12; MAGEA2B; MAGEA4; MAGEC1; MAGEC2; PAGE1; PAGE2; PAGES; PLAC1, PRAME; SPANKA1; SPANXB2; SPANXD; SSX1; SSX3; SSX4B, SSX7	ACTLB; CEP55; OIPS; PASD1; PBK; TMEFF2; TTK; CSAG2; CXorf48; GAGE3; GPAT2; LEMD1; LY6K; MAGEA1; MAGEA11; MAGEA6; MAGEB1; MAGEA11; MAGEA6; MAGEB1; PAGE2B; POTEB; POTEG; SSX2; ZNF16S		CASCS; CT47B1; DKKL1; GAGE1; LUZP4; NXF2; PAGE4; POTEC; POTED; POTEE; RGS22; ROCD1; SPA17; XAGE2B; XAGE3; XAGES	ACRBP; DPPA2; HSPB9; PIWIL2; SAGE1; SYCE1; TMEFF1; TSGA10; XAGE-4

Interferon, Antigen Presentation, Cytokine/Chemokine, Inflammation, and Influenza groups are categories of GSEA pathways. Percentages indicate how many genes from the GSEA gene set are included in AIM gene lists. “Common Genes in 3 Types of Cancer” lists the genes in each pathway upregulated by AZA in all three tumor types. “Common Genes in Any 2 Types” lists the genes in each pathway upregulated by AZA in any two cancer types. “Unique Genes” lists the genes in each pathway upregulated by AZA in only one tumor type.

Immune genes from these 15 common upregulated immune gene sets (Table 1) characterized by greater than twofold expression changes were then categorized as an AZA IMmune Gene set (AIM). The expression values for these AIM genes (Table 1), comprised of 317 genes from 63 cancer cell lines (breast, colorectal and ovarian) are shown arranged by the respective immune gene sets (Fig. S2). The plots detail the cell lines with the greatest gene expression changes in response to AZA and rectangles have been placed on these cell lines used for subsequent validation studies (Fig. S2).

The canonical effects of AZA have been described as demethylation of promoter regions and subsequent expression of the silenced gene [9, 10]. Many of the pathway changes in response to AZA, such as increased expression of immune genes, may be the result of downstream events elicited by a small number of hubs related to promoter DNA demethylation and associated gene upregulation [11]. We investigated hub networks in our current pan-cancer analyses by first searching, in a genome-wide analysis using the Infinium 450K methylation platform, for genes that have AZA-induced demethylation of cancer-specific, DNA hypermethylated, CpG islands associated with proximal promoter regions. The total number of such demethylated genes in the cell lines from breast, ovarian and colorectal cancers was 162 (Fig. S3a). A subset of these genes (4.9%) showed demethylation and reexpression in all three cancer types including *PYCARD*, *B3GALT4*, *CARD9*, *EID3*, *TSPYL5*, *IFF01*, *FERMT3*, and *AC5*. The highest percentages of these demethylation and reexpression events were again seen in immune genes; 26%, of the 162 genes were categorized as immune related (Fig. S3b,c). Overall immune gene demethylation and reexpression was again highest in ovarian cancer cell lines (53.8%) followed by colorectal (42.8%) and breast (30.7%) cancer cell lines (Figure S3b). Of note amongst these 162 genes, 8 (4.9%) were also in our AIM gene set (*BNIP3*, *HERC5*, *ICAM1*, *IRF7*, *MX1*, *MST1R*, *PSMB8*, *TCRIG1*) with *IRF7*, a member of the interferon regulatory factor family of transcription factors [5, 12] in particular being notable for being a canonical demethylated and reexpressed gene.

### Validation of AIM genes

**Figure 2:**
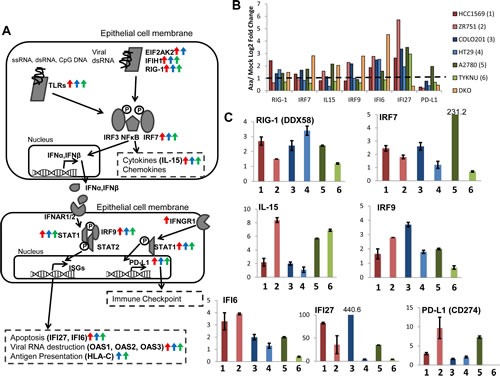
AZA activates diverse pathways involved in the immune response in breast, colorectal, and ovarian cancers A). Schematic of the interferon response to pathogens in an epithelial cell. Arrows next to gene names indicate that they are upregulated twofold by AZA in breast (red), colorectal (blue), or ovarian (green) cell lines. B) Upregulation of immune genes by AZA treatment in two cell lines from each tumor type (red = breast cancer, green = ovarian cancer, blue = colorectal cancer). Yellow bars denote the fold change of the DKO cell line (haploinsufficient for DNMT1 and null for DNMT3) compared to the parent HCT116 cell line. Y-axis represents AZA/Mock fold change (log2). C) qRT-PCR validations of genes from 2B. Y-axis represents AZA/Mock fold change (linear). Cell lines are the same colors as in 2B. Each bar represents the average and standard deviation of three biological replicates.

In order to validate our findings for AIM genes from the expression microarrays, we investigated selected genes by quantitative reverse transcriptase PCR (qRT-PCR) in two cell lines from each cancer type which showed the highest upregulation of transcripts in response to AZA in the array (HCC1569 and ZR751 for breast cancer, COLO201 and HT29 for colorectal cancer, and A2780 and TYKNU for ovarian cancer) (Fig. S2). We concentrated on key genes for individual steps in the associated immune pathways and especially for the interferon response as selected by the array data (Fig. 2a, b). Many AIM genes are part of or downstream of the interferon response (including antigen presentation and cytokines/chemokines) [13]. Each chosen gene validated in the qRT-PCR assays for AZA-induced reexpression (Fig. 2c).

**Figure 3:**
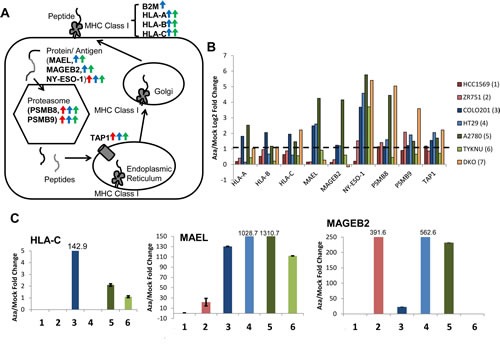
AZA activates genes involved in antigen presentation and processing in breast, colorectal, and ovarian cancers A) Schematic of antigen processing. Arrows next to gene names indicate that they are upregulated twofold by AZA in breast (red), colorectal (blue), or ovarian (green) cell lines. B) Upregulation of antigen presentation genes by AZA treatment in two cell lines from each tumor type (red = breast cancer, blue = colorectal cancer, green = ovarian cancer). Yellow bars denote the fold change of the DKO cell line (haploinsufficient for DNMT1 and null for DNMT3) compared to the parent HCT116 cell line. C) qRT-PCR validations of genes from 3b. HLA-C was undetectable by qRT-PCR in HCC1569, ZR751, and HT29. Each bar represents the average and standard deviation of three biological replicates.

GSEA analysis identified antigen processing and presentation as key pathways upregulated by AZA (Fig. 1c, Fig. 3a); these are among the interferon regulated genes in the type I interferon response [14]. Antigens and antigen presentation genes were upregulated in representative cell lines from each tumor type and in DKO cells (Fig. 3b). Upregulation of selected genes was validated by qRT-PCR (Fig. 3c) and represent regulation by AZA at most every step of antigen presentation, in all three cancer types (Fig. 3a).

It is especially noteworthy that the DKO cell line (haploinsufficient for DNMT1 and null for DNMT3B), which is shown as a genetic mimic of AZA treatment (Fig. 2b, Fig. S2) induces significant upregulation for most AIM genes (Fig. S4a,c). To determine whether this was specific to DNMT inhibitors, we also treated cells with an HDAC inhibitor (TSA) that has been used extensively in our laboratory. We show that TSA also upregulates subsets of AIM genes but not as uniformly or robustly as DKO cells or AZA treated cells (Fig. S4a,b,c). This activation of AIM genes appears to be in response to epigenetic agents and not the result of a general cell stress response that could be elicited by chemotherapeutics such as carboplatin. Treatment of the ovarian cancer cell line A2780, for 72 hours with 500 nM carboplatin does not lead to overexpression of AIM genes *IFI27*, *IRF7*, *IL15*, or *MAGEB2*, all of which are increased in AZA-treated cells (Fig. S4d).

Demethylation and upregulation of cancer testis antigens by AZA has been previously described [15-18]. Cancer testis antigens are critical to tumor immunology, but GSEA does not have a well-defined cancer testis antigen gene list. Thus we created a gene set from the well-annotated CTdatabase [19] and ran GSEA on the 63 cell lines using the same cutoffs for significance as in Figure 1. The cancer testis antigens were significantly enriched in many cell lines, and were only upregulated by AZA (Fig. S5). The upregulation of cancer testis antigens was again seen in all three cancer types although this was more pronounced for colorectal (64.3% of cell lines) and ovarian (39.1%) and less so for breast (19.2%) cancers.

### AIM Gene Signature in Primary Cancers

**Figure 4:**
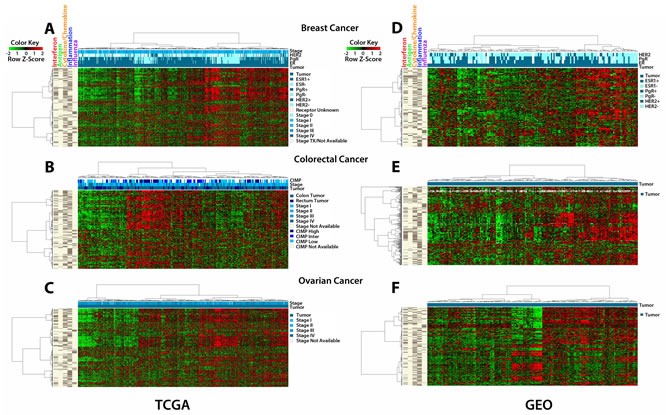
The AIM 317 gene panel clusters TCGA and GEO tumors into high and low immune signatures Tumors from The Cancer Genome Atlas (TCGA) cluster into “high” and “low” immune groups based on the AIM genes. The bars on the far left show the five sets of AIM genes driving the clustering, interferon, antigen, cytokines/chemokines, inflammation and influenza. The shades of blue bars at the top denote tumor vs. normal, stage, and receptor status for breast cancer, CIMP, stage, and colon vs rectum for colon/rectum cancer, and stage for ovarian cancer. The heat map shows transcript levels from green (low) to red (high). A) breast cancers; B) colorectal cancers; C) ovarian cancers. Tumors from publicly available (GEO) data sets show similar clustering: D) breast cancers; E) colorectal cancers; F) ovarian cancers.

It is critical to know how all of the above work performed in cultured cancer cells may relate to primary cancers. We thus examined how basal levels of the AIM genes might reveal clustering of hundreds of primary samples in publicly available gene expression data sets from breast [20], colorectal [21], and ovarian [22] cancers in The Cancer Genome Atlas (TCGA) and the Gene Expression Omnibus (GEO) (Fig. 4) (TCGA datasets included 536 breast, 155/69 colon/rectal and 590 ovarian cancers, and for GEO the breast, colorectal and ovarian datasets contained 177,188 and 185 cancers, respectively). Significantly, each cancer type, in each database, clustered into sub-groups that have very concordant “low” or “high” expression of the 317 AIM genes (Fig. 4). For TCGA data, no correlation was observed with clinical stage or tumor subtype in either breast, colorectal or ovarian cancers (Fig. 4a,b,c). These clinical parameters were less well defined in GEO. We also did not see an association of AIM gene expression with breast cancer subtype (ER+, HER2+, triple negative) (Fig. 4a,d). Because of the smaller number of colon cancers in the TCGA, both colon and rectal cancer expression data were combined for the AIM analysis and we found that there was no distinct cluster associated with either tissue type (colon or rectal). However, higher AIM gene expression did appear to associate with a large percentage of colorectal tumors that had a high CIMP status [23] (Fig. 4b).

The low basal levels of the AIM genes in primary cancers of all three types suggests what has been termed a cancer immune evasion phenotype [24, 25], which can be reversed by AZA treatment. Our previous data in NSCLC with a less comprehensively annotated gene set had also suggested this [5]. We thus examined our AIM gene panel in the TCGA data set for NSCLC. Remarkably, TCGA expression data from lung cancers showed similar clustering of AIM gene sets into a “high” and “low” expression cluster (Fig. S6a). We examined our AIM profile in the TCGA melanoma database since excitement over targeting immune tolerance for solid tumors has been particularly high for this disease. Again, an intriguing clustering of AIM gene sets into a “high” and “low” expression cluster is seen (Figure S6b).

**Figure 5:**
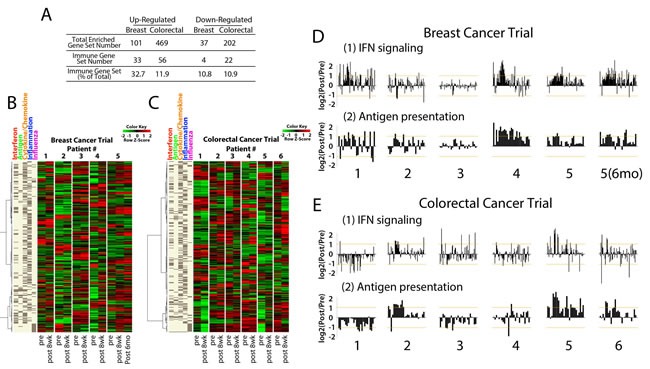
Core biopsies from breast and colorectal cancer patients treated with AZA/entinostat show upregulation of the AIM genes A) Summary of GSEA gene sets upregulated and downregulated by AZA/entinostat in breast and colorectal cancer biopsies. Percentages of gene sets that are immune-related are listed. Heat maps for B) triple negative breast and C) colorectal cancer trial samples. Each pair includes “Pre” (baseline or before AZA/entinostat treatment) and “Post” = 8 weeks after AZA/entinostat treatment) and depicts levels of AIM genes (listed on the left). D-E). Bar plots for each breast cancer (D) or colorectal cancer (E) patient biopsy represent a log2 (Pre/Post) fold change (y axis) of individual genes in the GSEA interferon signaling and antigen presentation gene sets. Breast cancer patient #5 6 mo) represents the 6 month post biopsy specimen.

To address the question of whether AIM genes are re-expressed *in vivo*, we queried RNA from patients with triple negative breast cancer [26] and colorectal cancer receiving combination epigenetic therapy with AZA and an HDAC inhibitor, entinostat, with the AIM panel. We examined biopsies obtained from patients pre- and post- (8-weeks) epigenetic therapy. GSEA analysis of expression data from paired patient biopsies revealed that 32.7% (33/101) of the GSEA gene sets upregulated in breast cancers were immune related while colorectal cancers contained 11.9% (56/469) upregulated immune gene sets (Fig. 5a). Strikingly, of the 15 common upregulated immune gene sets from the 63 AZA treated cancer cell lines (Fig. 1c), 11 immune gene sets were upregulated in biopsies from both breast and colorectal patients after 8 weeks of therapy. The 317 AIM genes derived from our cell line experiments were used to query the expression data from the paired biopsies, and AIM genes were upregulated by AZA in the post treatment tissue (Fig. 5b,c). For example, breast cancer patient #5 showed increased expression of AIM genes at 8 weeks of AZA/entinostat therapy and this increase was maintained, if not increased, in a 6 month biopsy (Figure 5b). This patient showed significant fold change expression for the interferon signaling (α/β and γ) gene sets in the AIM panel (Fig. 5d). Similarly breast cancer patients 1 and 4 also showed strong increases in the AIM gene panel and again for interferon signaling expression in response to combination epigenetic therapy with AZA and entinostat (Fig. 5b,d). Colorectal cancer patients 2, 5 and 6 showed increases in AIM gene expression in the 8 week post biopsy (Fig. 5c) especially for individual AIM gene sets such as antigen presentation (Fig. 5e).

## DISCUSSION

In this study, we investigated a response to an epigenetic agent, the DNA demethylating drug AZA, in three common solid tumors. This is an important issue because AZA is FDA approved for MDS, and given at low doses which preserve on-target effects and minimize off-target ones, its promise for efficacy in solid tumors is emerging [4-6, 11]. In our preclinical studies of cell lines from breast, colorectal, and ovarian tumors, transient, low-dose AZA alters many pathways key for tumorigenesis including cell cycle and mitotic pathways [27, 28], mRNA splicing and translation [29], transcription and DNA replication [27, 28]. However, the dominant effect was an upregulation of immunomodulatory pathways. The importance of these findings to the emerging possibility of a role for epigenetic therapy for sensitizing patients with cancer to immunotherapy has been stressed throughout our manuscript.

Importantly, we have highlighted two ways in which our cell culture data have key relationships to primary tumors for not only the three cancer types studied but also for NSCLC and melanoma. First, the AIM gene panel we have generated clusters basal expression levels for hundreds of primary samples across five tumor types and multiple expression databases into high and low immune gene expression groups. With the close relationship of the involved genes to key immune pathways such as interferon responses to inflammation, viral challenge, *etc.*, low levels of the AIM genes represent cancers with what has been termed an immune evasion signature [24, 25]. In fact, previous studies have described immune enriched subtypes in several types of solid tumors including triple negative breast cancer [30], colon cancer [31], and an “immunoreactive subtype” of serous ovarian carcinoma [32].

Taken together, these data show that solid tumors can be described as immune low or immune enriched and suggests that patients with low expression of immune AIM genes might benefit most from receiving epigenetic therapy prior to immunotherapy. Our pan-cancer data would, then, provide a rich roadmap for a biomarker strategy that might track, and help personalize, such a scenario. Second, for the above biomarker implications, although the patient numbers are low and immunotherapy was not given, we have provided evidence that genes in our AIM panel are upregulated by epigenetic therapy in patient tumor biopsy samples for two of the cancer types studied, breast and colorectal cancer.

A question that remains to be answered in our study is the role that AZA plays in regulating the observed changes in gene expression signatures. Classically, this drug blocks DNA methylation, and this could lead to re-expression of promoter methylated and silenced genes [1]. While we believe this certainly is contributing to the immune response observed, most of the AIM genes do not have canonical CpG island promoters.

Many key pathway changes for anti-tumor responses, and perhaps most gene expression changes including AIM genes, may lie downstream of a hub triggered in a cancer cell by classic promoter demethylation. Furthermore, for the low AZA doses employed, we see significant overall DNA demethylation (Fig. S3) and specific events for key genes in our immune pathways (Fig. S3). A key example in this work with high correlation to AIM gene responses and to events in the interferon pathway in our previous study of NSCLC [5] is the transcription factor gene, *IRF7*.

This will especially hold true for the low doses of AZA that are used in clinical trials with epigenetic therapy [11]. Low doses of AZA which we have shown are effective in solid tumors [6] may not re-express all densely hypermethylated genes as high doses of demethylating agents can. Interestingly, most of the immune genes in our AIM panel do not have CpG island promoters and the epigenetic mechanism controlling their re-expression is not clear. However the increase in gene expression could be related to the scaffolding actions of DNMT1 and how AZA-induced degradation of this methyltransferase could affect the binding of other chromatin regulators, thereby leading to chromatin remodeling and increased transcription [33]. The targeted role of AZA on degrading DNMTs is highly reflected in the very similar responses of these AIM genes to genetic depletion of DNMTs in the DKO cells (Fig. S4).

Our preclinical studies using AZA initially derived the AIM gene panel from cultured epithelial cancer cells, and although it seems likely that the increased immune signature in patient biopsies treated with AZA/entinostat is coming from the tumor cells, the immune signature may also be influenced by drug effects on stroma, and infiltrating immune cells. HDAC inhibitors have been shown to have effects on the host immune system [34]. Our preclinical TSA data shows that in epithelial cells HDAC inhibitors also regulate a significant number of immune genes. A compelling question remains about the relative contributions of each drug type to regulation of gene expression in epithelial versus host immune cells. These results suggest why a combination of AZA and an HDACi, as used in our ongoing NSCLC trials [3, 5], may be an optimal approach in the clinic.

Our current findings showing a universal upregulation of immune genes by epigenetic drugs in multiple solid tumor types indicate a strong immunomodulatory role for these drugs in cancers. Our derived AIM gene panel identifies the subset of patients with a low basal immune gene expression signature that may derive the greatest benefit from epigenetic priming for immune therapy.

## METHODS

### Cell Line Treatments

63 cell lines (26 breast cancer, 14 colorectal cancer, 23 ovarian cancer) were used in these experiments. Breast cell lines included BT20, BT474, CAMA1, EFM19, MDA453, MDA468, MDA361, MCF7, MDA231, T47D, HCC1500, and HCC1187 obtained from the American Type Tissue Collection; HCC1419, HCC38, EFM192A, HCC1569, HCC1937, HCC1954, MDA175, MDA415, MDA436, SUM149, SUM159, SKBR3, ZR751, and ZR7530 from Dr. Dennis Slamon. All cells were maintained under recommended conditions. Colorectal cell lines were all obtained from the American Type Tissue Collection and were maintained under recommended conditions. These included CACO-2, Colo201, Colo205, Colo320, DLD1, HCT116, HT29, Lovo, RKO, SK-CO1, SNUC-1, SW48, SW480, and SW620. Ovarian cell lines were obtained from the laboratory of Dr. Dennis Slamon and included A2780, CAOV3, DOV13, EFO27, ES2, Hey, HEYC2, Kuramochi, OAW28, OAW42, OV167, OV2008, OV90, OVCA429, OVCA432, OVMANA, OVCAR3, OVCAR5, OVKATE, PEO14, SKOV3, TOV112D, and TykNu; these were maintained under the ATCC recommended conditions.

Cell lines were treated with 500 nM of AZA or carboplatin (Sigma; St. Louis, Missouri) for 72 hours while in log-growth phase, changing the media and drug every 24 hours for AZA treatment. To select an appropriate chemotherapy control, the carboplatin dose that had the similar growth inhibitory effect to 500 nM AZA after 10 days was used to treat the cells. Cells were harvested at 1, 3, 7, 10, 14, 21, or 28 days following initial application of drug. DNA and RNA were obtained using standard protocols [6]. RNA from 63 cell lines was sent for the Agilent 44K Expression Array and DNA from 53 cell lines was sent for the Illumina 450K Methylation Array [6].

### Clinical Trials

Patients were recruited to clinical trials NCT01349959 (breast cancer) and NCT01105377 (colon cancer). Patients received 40 mg/m^^2^ 5-azacitidine subcutaneously on days 1-5 and 8-10 and 7 mg oral entinostat on days 3 and 10. Courses were repeated every 28 days in the absence of disease progression or unacceptable toxicity. RNA was isolated from pre- (baseline) and post-treatment (8 weeks) biopsies and analyzed with the Agilent 44K Expression Array.

### Bioinformatics

All data were analyzed using R: A Language and Environment for Statistical Computing [35]. Expression normalization of cell line data was performed using the package Limma as previously described [9,36]. Data was normalized within each tumor type (breast, colorectal, and ovarian). These normalized values were then analyzed utilizing Gene Set Enrichment Analysis by the Broad Institute and data packages (C5BP, Reactome, KEGG) [7]. Pathways enriched with a false discovery rate less than 0.25 and normalized enrichment score (NES) > 2.15 (upregulated gene sets), or <−2.15 (downregulated gene sets) were chosen. These criteria represented the ~top 30% of all upregulated gene sets as determined by the NES score. Pathways common among breast, colorectal, and ovarian cancer were identified. Pathways were manually curated into specific categories. AIMs were defined by intersection of all genes from the enriched GSEA gene sets with over 2 fold upregulated genes after AZA treatment for any cell line, any time point. Genes were defined as demethylated if they met the following criteria: had a high basal β value > 0.5 and a Δβ _(AZA-Mock)_ <−0.25, were expressed at low basal levels in the untreated cells (lower than 50% of the expression quantile) and expressed at higher levels in the AZA treated cells (> 2-fold). For β values, the only gene probes included in the analysis were those that recognized the CpG island within the promoter. Demethylated/re-expressed genes had to meet both demethylation and reexpression criteria at least in one cell line. TCGA HumanMethylation27K level 3 data was downloaded, standard deviation of Infinium β-values across all primary cancer samples were calculated, and the top one thousand most variable probes were chosen for hierarchical cluster analysis [23]. Based on the dendrogram and overall methylation status, primary cancer samples were classified as CIMP high, CIMP intermediate and CIMP low.

### Validations (qRT-PCR)

After total cellular RNA was extracted using the Trizol method (Life Technologies, Carlsbad, California), RNA concentration was determined using the Nanodrop machine and software (Thermo Fisher Scientific, Rockville, Maryland). 1 μg total RNA was used to generate cDNA with the QuantiTect Reverse Transcription Kit (Qiagen, Venlo, the Netherlands). Quantitative reverse transcription PCR (q-RT-PCR) of CD274, DDX58, HLA-C, IFI6, IFI27, IL-15, IRF7, IRF9, MAEL, and MAGEB2 mRNA was performed using TaqMan assays (Life Technologies, Carlsbad, California) and the Applied Biosystems 7500 Fast real-time PCR system and software. Human β-actin mRNA was used as the endogenous control [37]. The ΔΔCT method was used to calculate relative expression levels. All qRT-PCR assays were carried out in triplicate and then repeated with new cDNA synthesis. Minus RT controls (reverse transcriptase negative cDNA synthesis reactions) were performed for at least one sample per plate.

## SUPPLEMENTARY FIGURES AND TABLES




