# Supplementary material for: Immune regulation by low doses of the DNA methyltransferase inhibitor 5-azacitidine in common human epithelial cancers
Source: Oncotarget. 2014 Feb 16;5(3):587–98. doi: 10.18632/oncotarget.1782 (PMC3996658; doi:10.18632/oncotarget.1782)
Supplement: Supplementary file 1 [file oncotarget-05-587-s001.pdf]

Immune regulation by low doses of the DNA methyltransferase inhibitor 5-azacitidine in common human epithelial cancers – Li et al

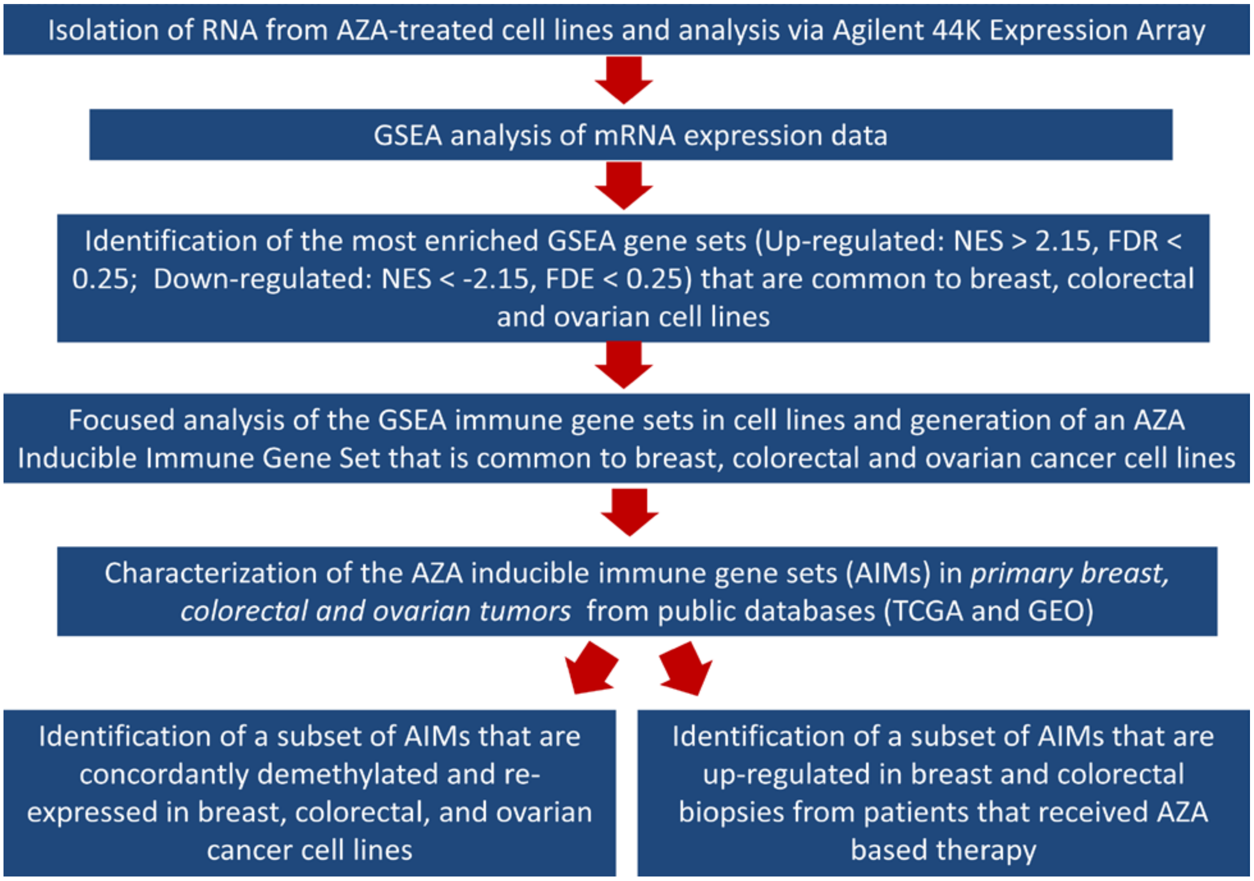

Figure S1: Schematic of analysis of AZA-treated cell lines and generation of AIM gene panel. Agilent array data were normalized and analyzed by GSEA. The most enriched GSEA gene sets for each tumor type were intersected to produce 80 common upregulated gene sets, out of which we focused our analysis on the upregulated immune gene sets. This immune signature was applied to primary tumors from publicly available cohorts as well as biopsies from AZA and entinostat trials in breast and colorectal cancer.

Figure S2A

RESPONSE_TO_WOUNDING AIM Genes

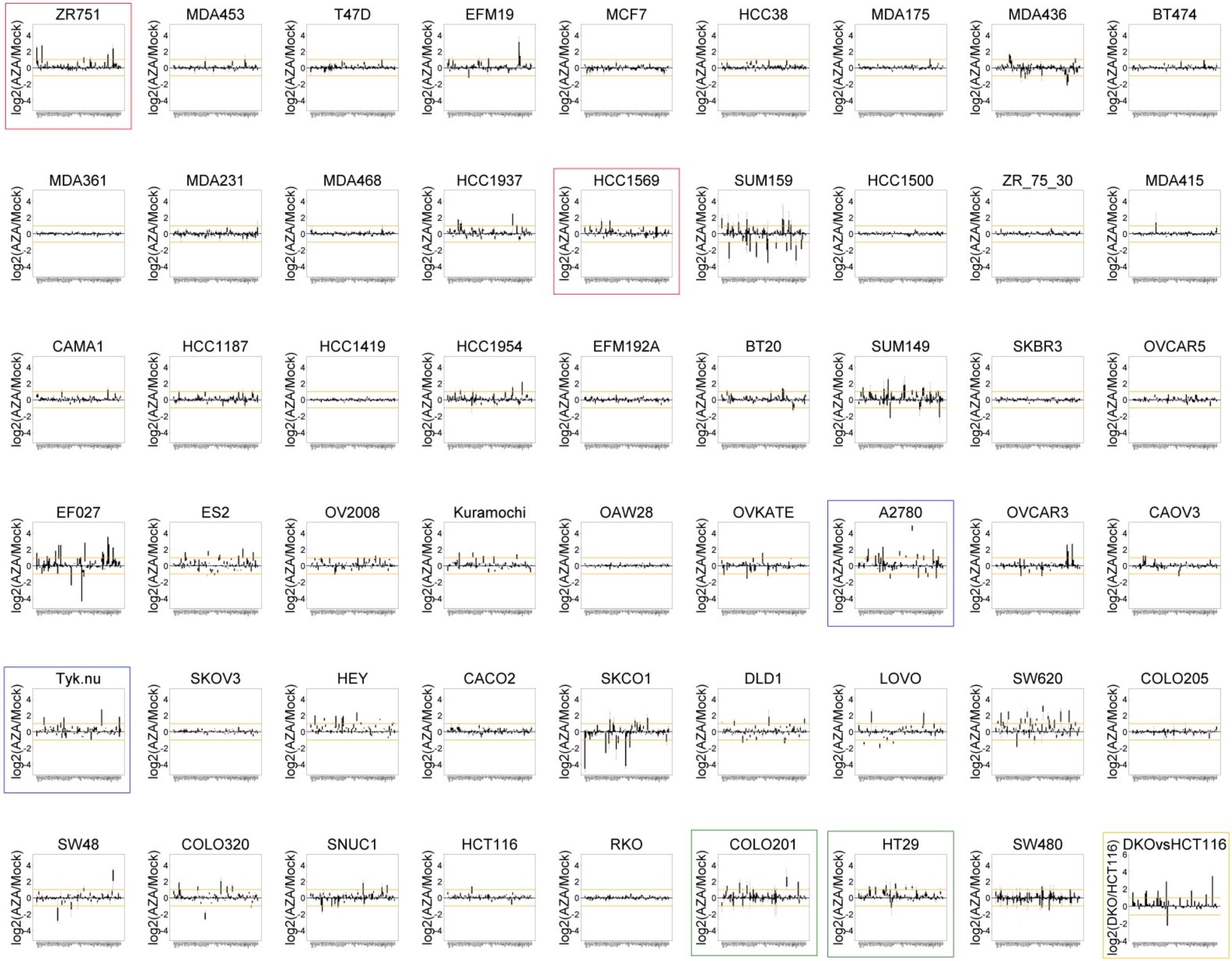

Figure S2B

REACTOME_NEGATIVE_REGULATORS_OF_RIG_I_MDA5_SIGNALING AIM Genes

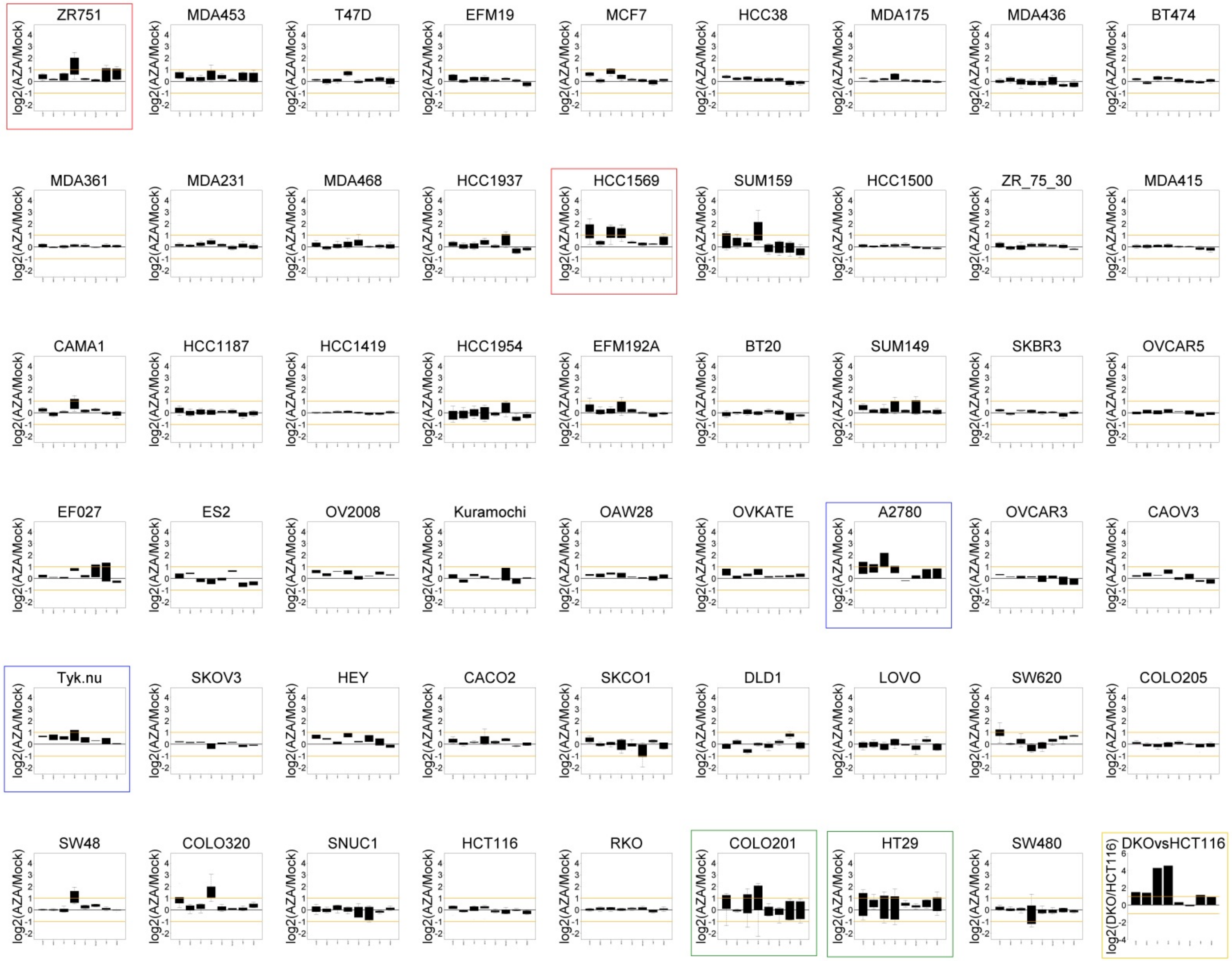

Figure S2C

REACTOME_INTERFERON_SIGNALING
AZA AIM Genes

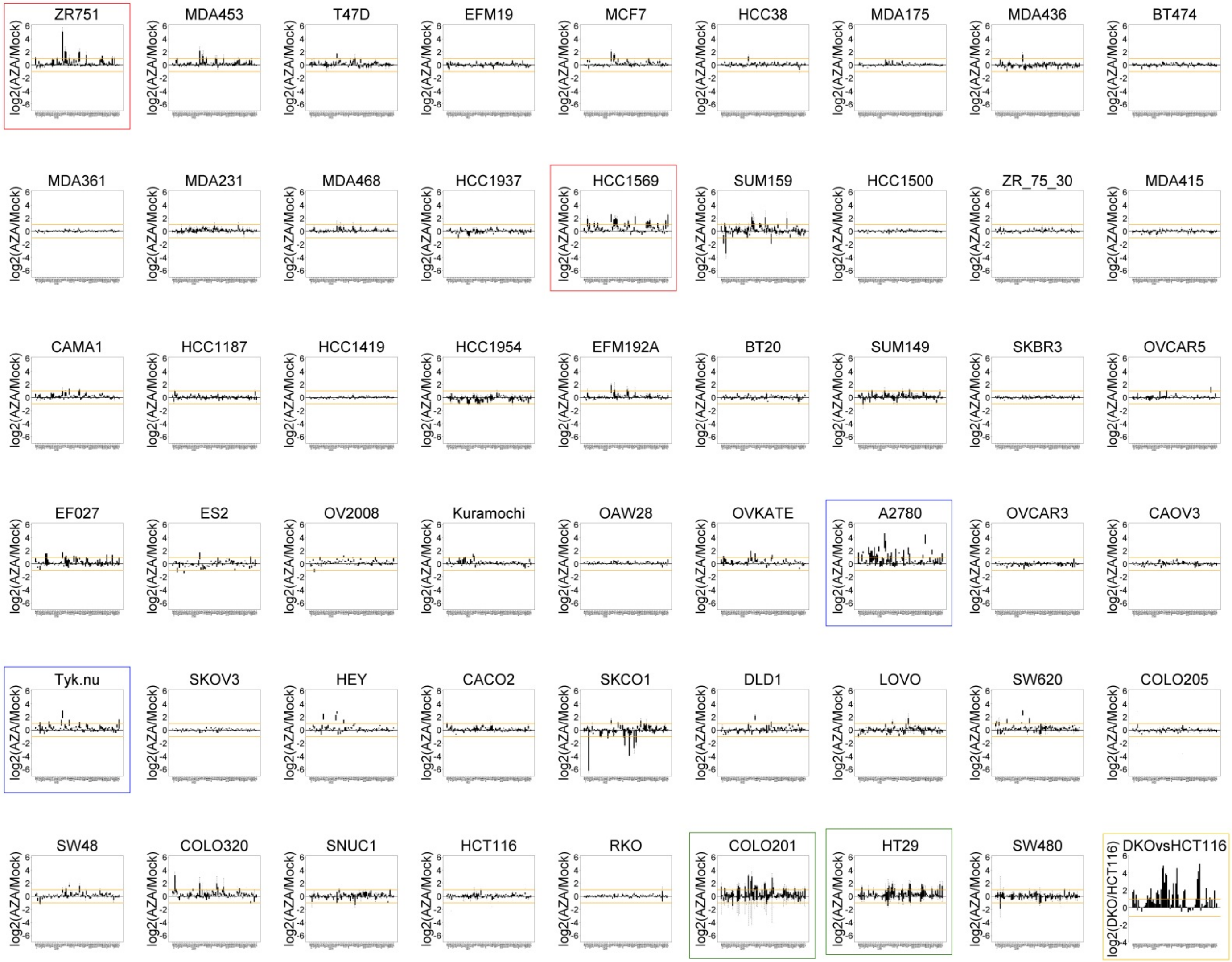

Figure S2D

REACTOME_INTERFERON_GAMMA_SIGNALIN G AZA AIM Genes

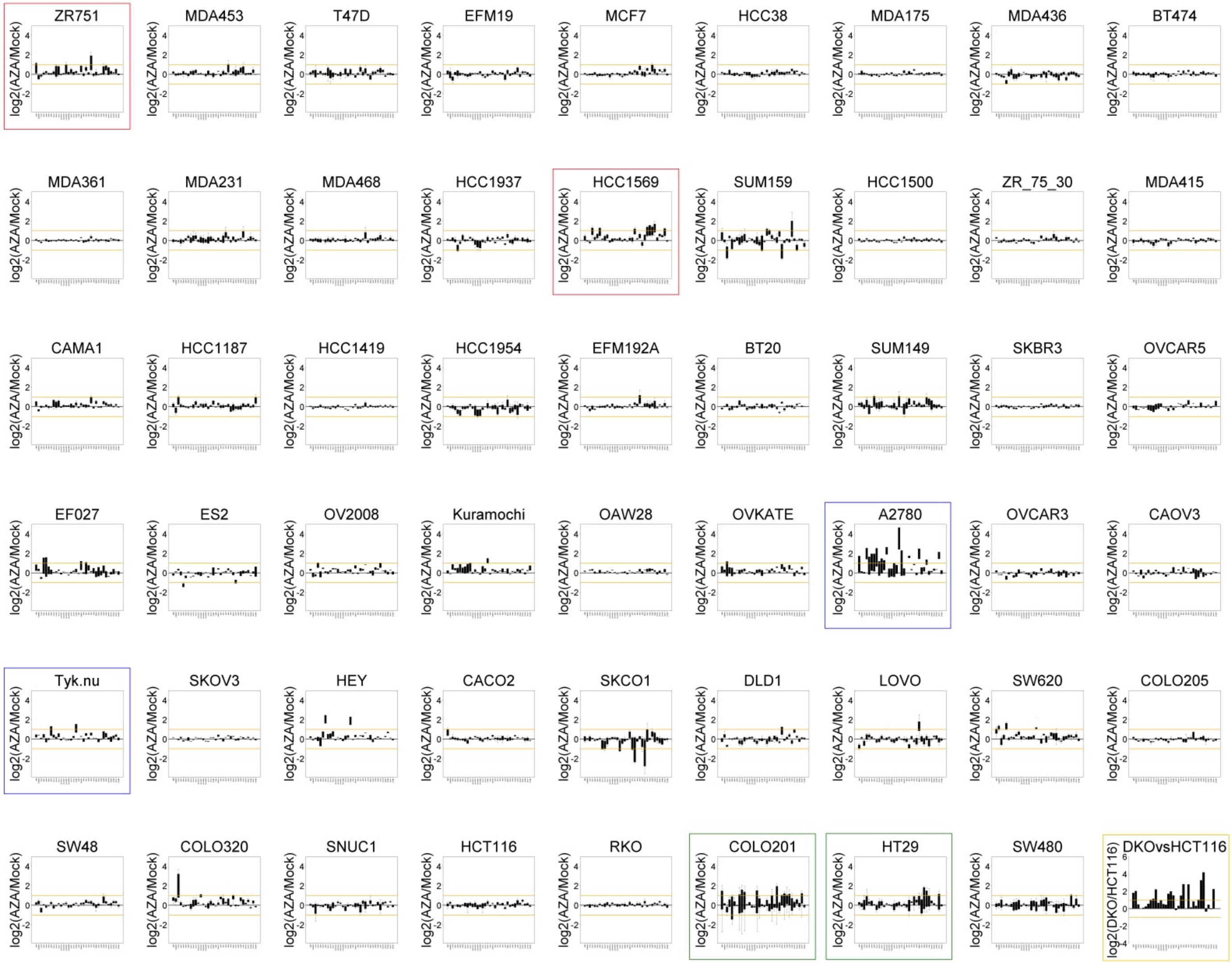

Figure S2E

REACTOME_INTERFERON_ALPHA_BETA_SIGNALING AZA AIM Genes

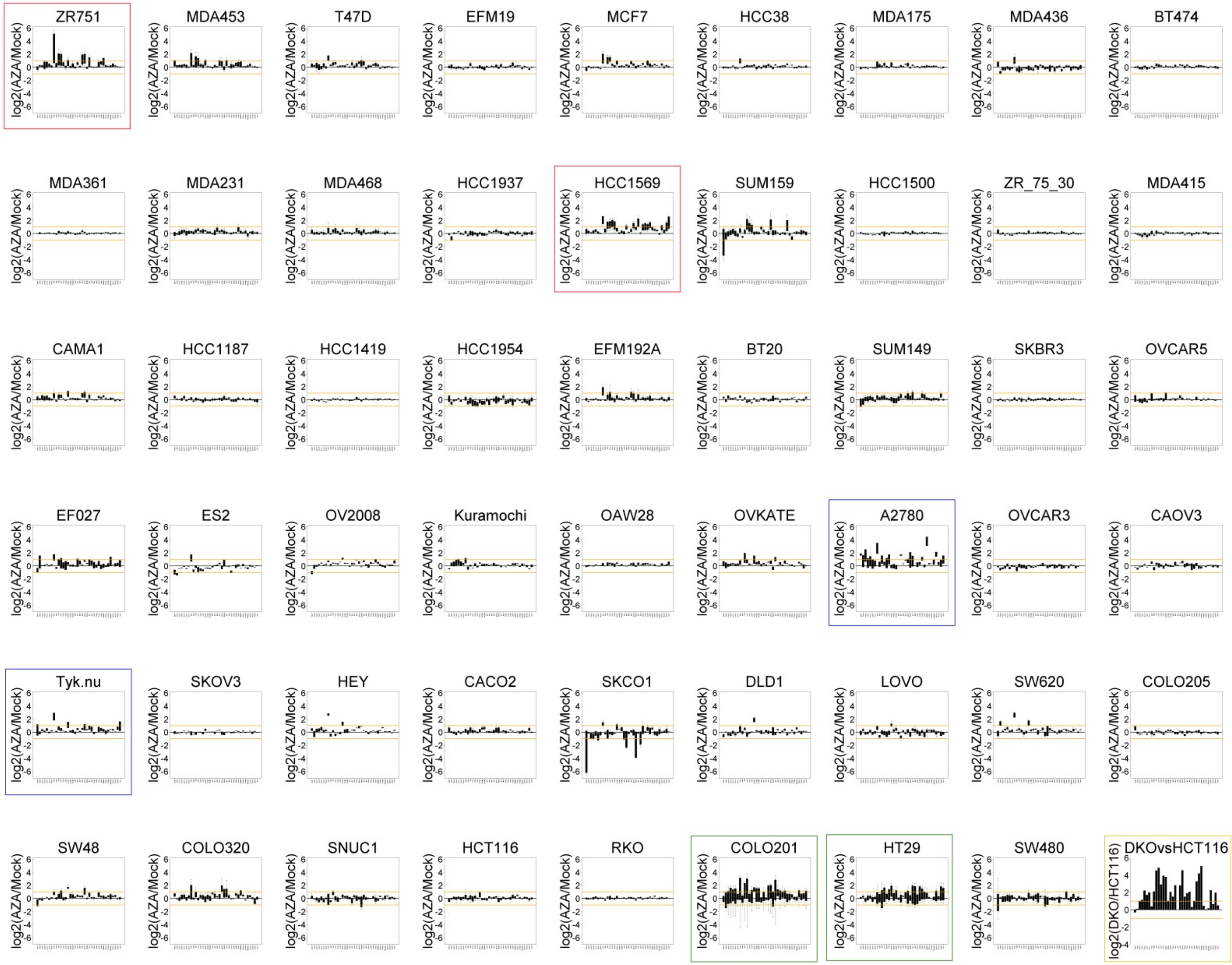

Figure S2F

REACTOME_INFLUENZA_VIRAL_RNA_TRANSCRIPTION_AND_REPLICATION AIM Genes

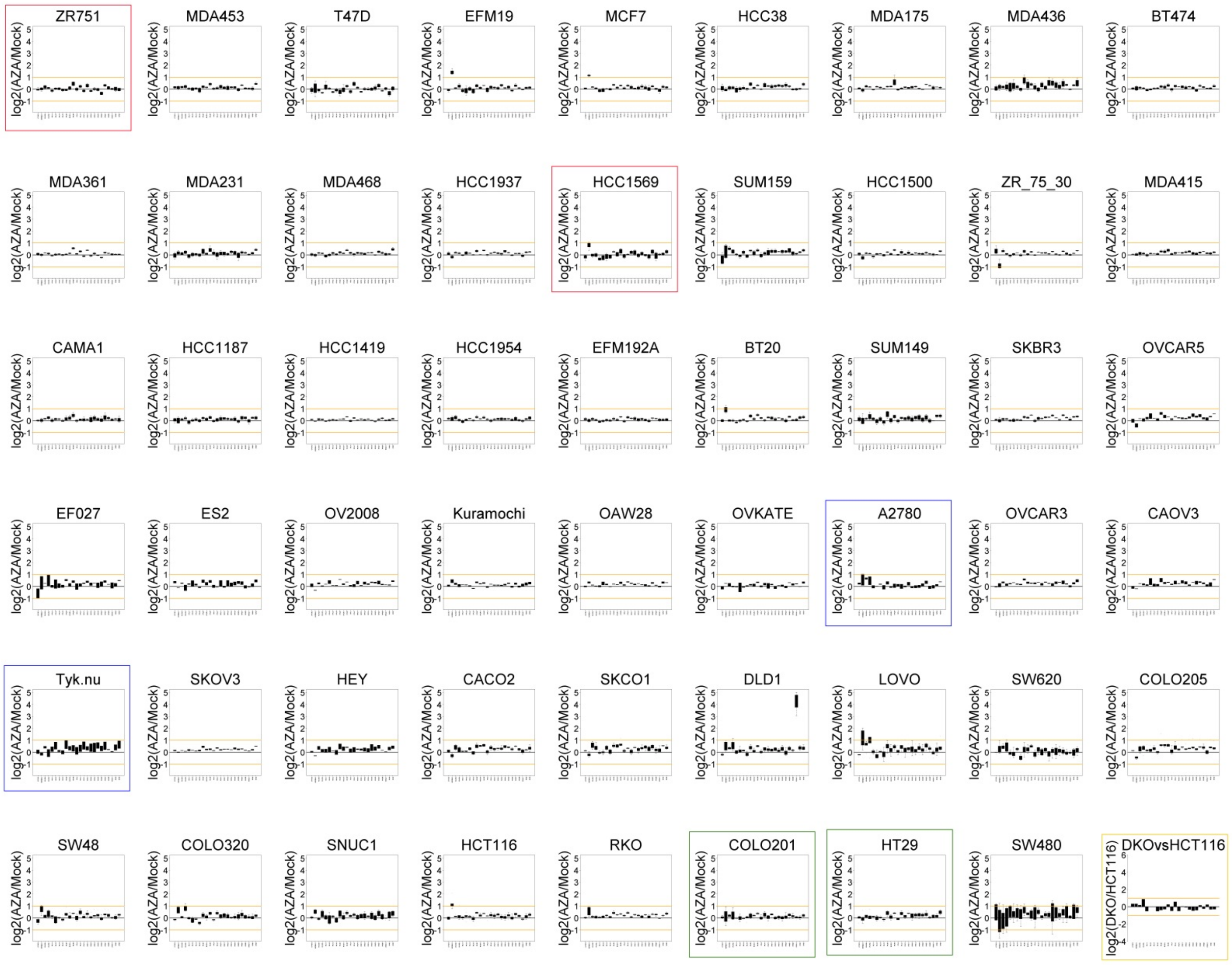

Figure S2G

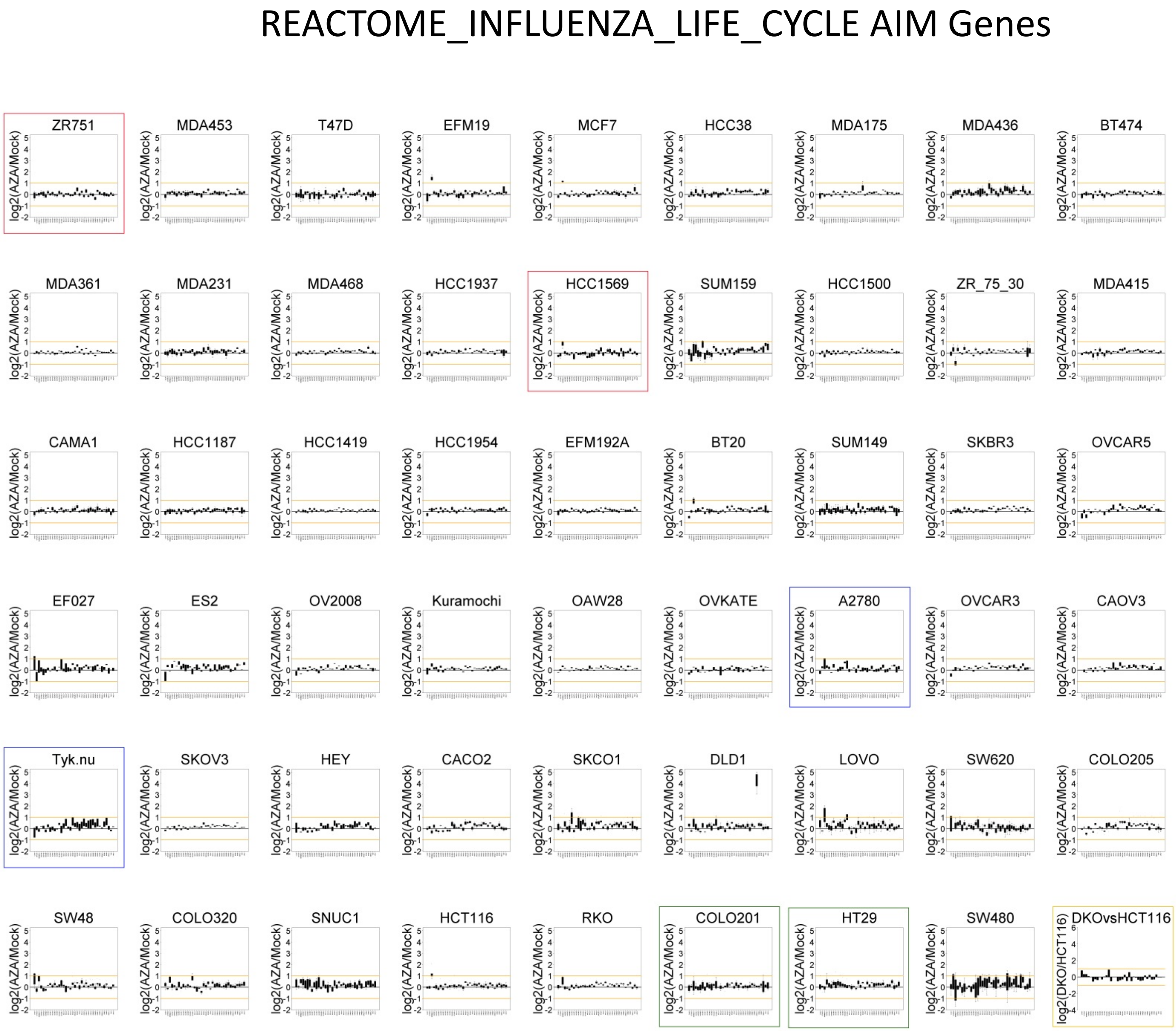
REACTOME_INFLUENZA_LIFE_CYCLE AIM Genes
ZR751
MDA453
T47D
EFM19
MCF7
HCC38
MDA175
MDA436
BT474
MDA361
MDA231
MDA468
HCC1937
HCC1569
SUM159
HCC1500
ZR_75_30
MDA415
CAMA1
HCC1187
HCC1419
HCC1954
EFM192A
BT20
SUM149
SKBR3
OVCAR5
EF027
ES2
OV2008
Kuramochi
OAW28
OVKATE
A2780
OVCAR3
CAOV3
Tyk.nu
SKOV3
HEY
CACO2
SKCO1
DLD1
LOVO
SW620
COLO205
SW48
COLO320
SNUC1
HCT116
RKO
COLO201
HT29
SW480
DKOvsHCT116
log2(AZA/Mock)
log2(DKO/HCT116)

Figure S2H

REACTOME_ER_PHAGOSOME_PATHWAY AIM Genes

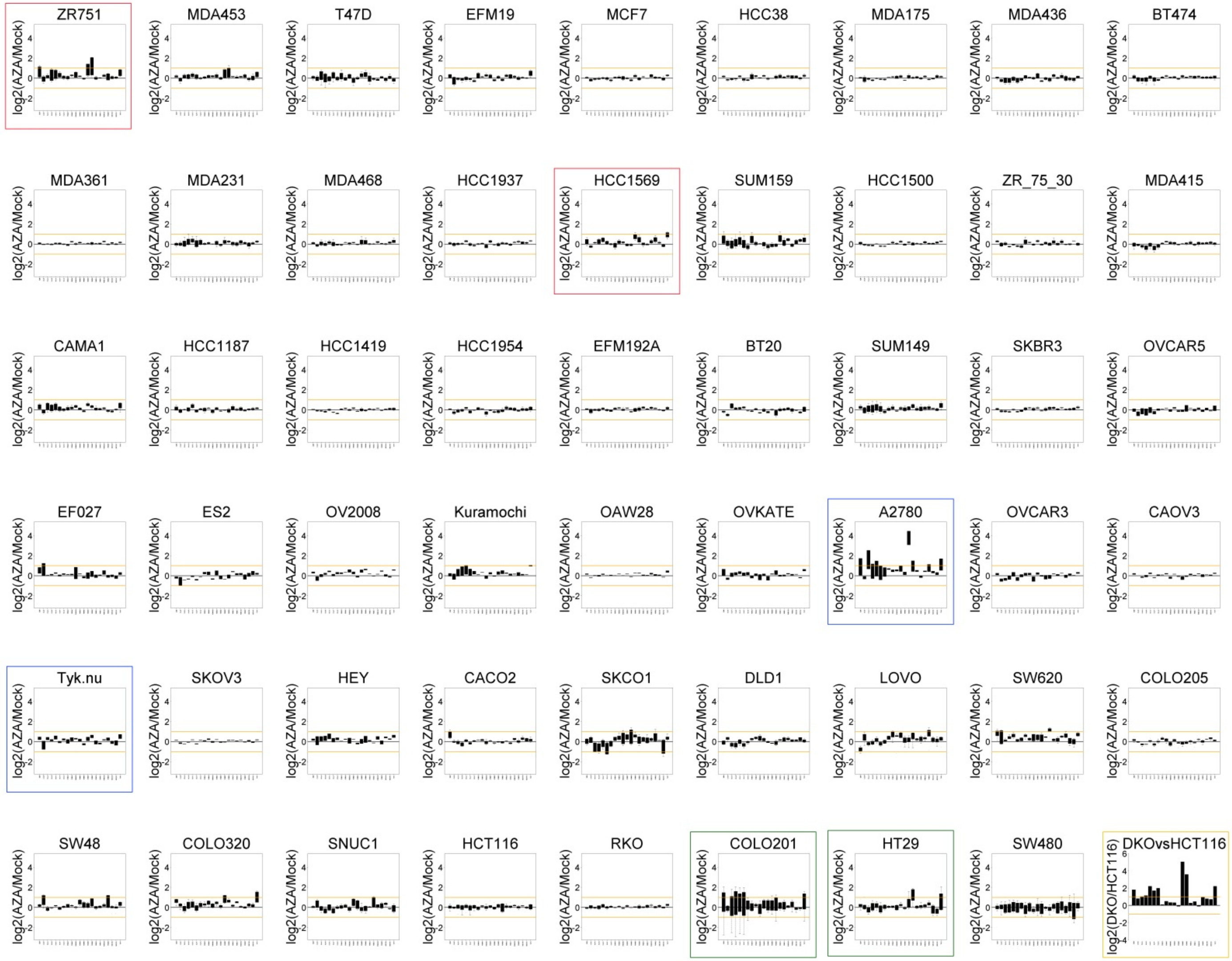

Figure S2 I

REACTOME_CYTOKINE_SIGNALING_IN_IMMU
NE_SYSTEM AIM Genes

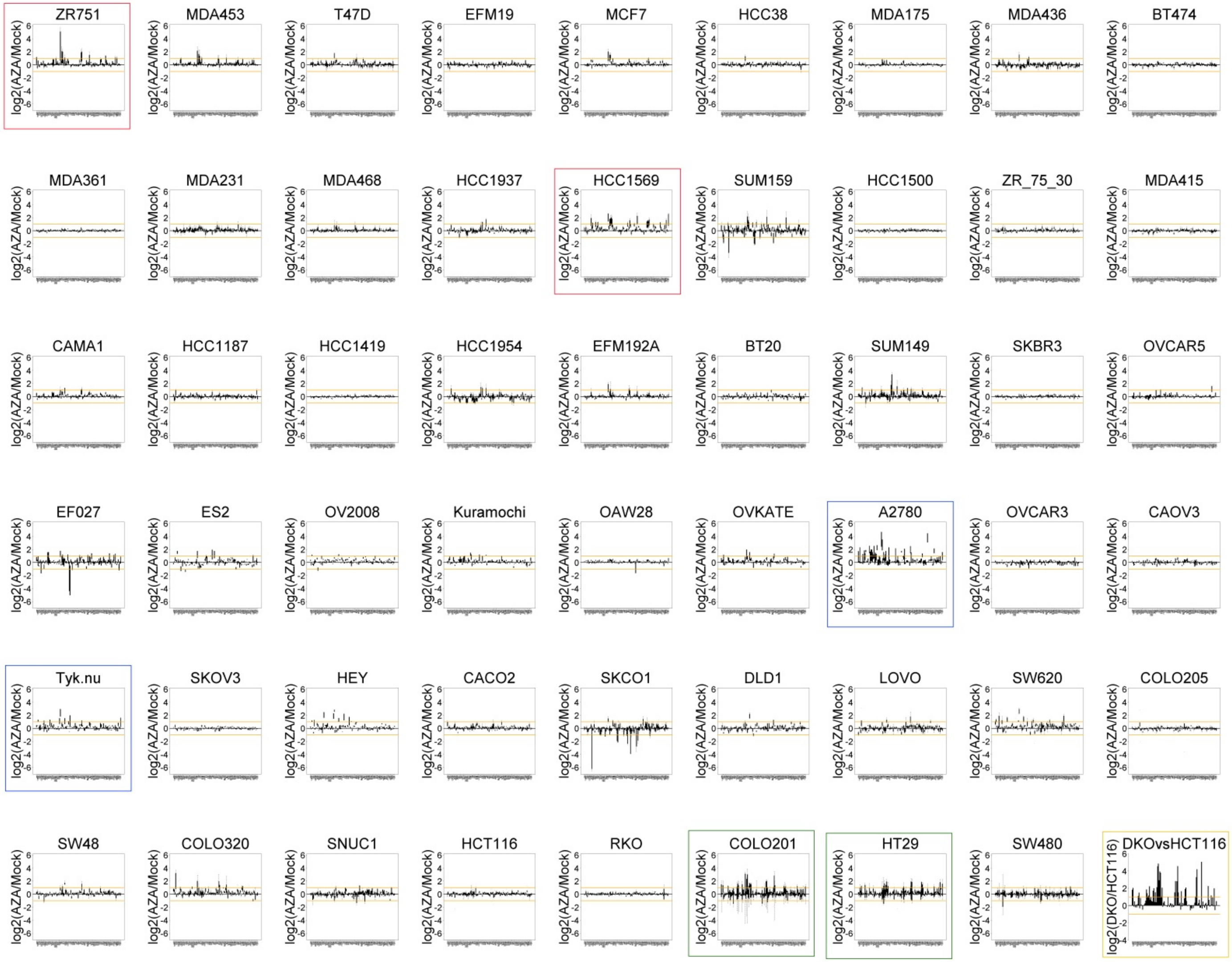

Figure S2 J

REACTOME_CHEMOKINE_RECEPTORS_BIND_C HEMOKINES AIM Genes

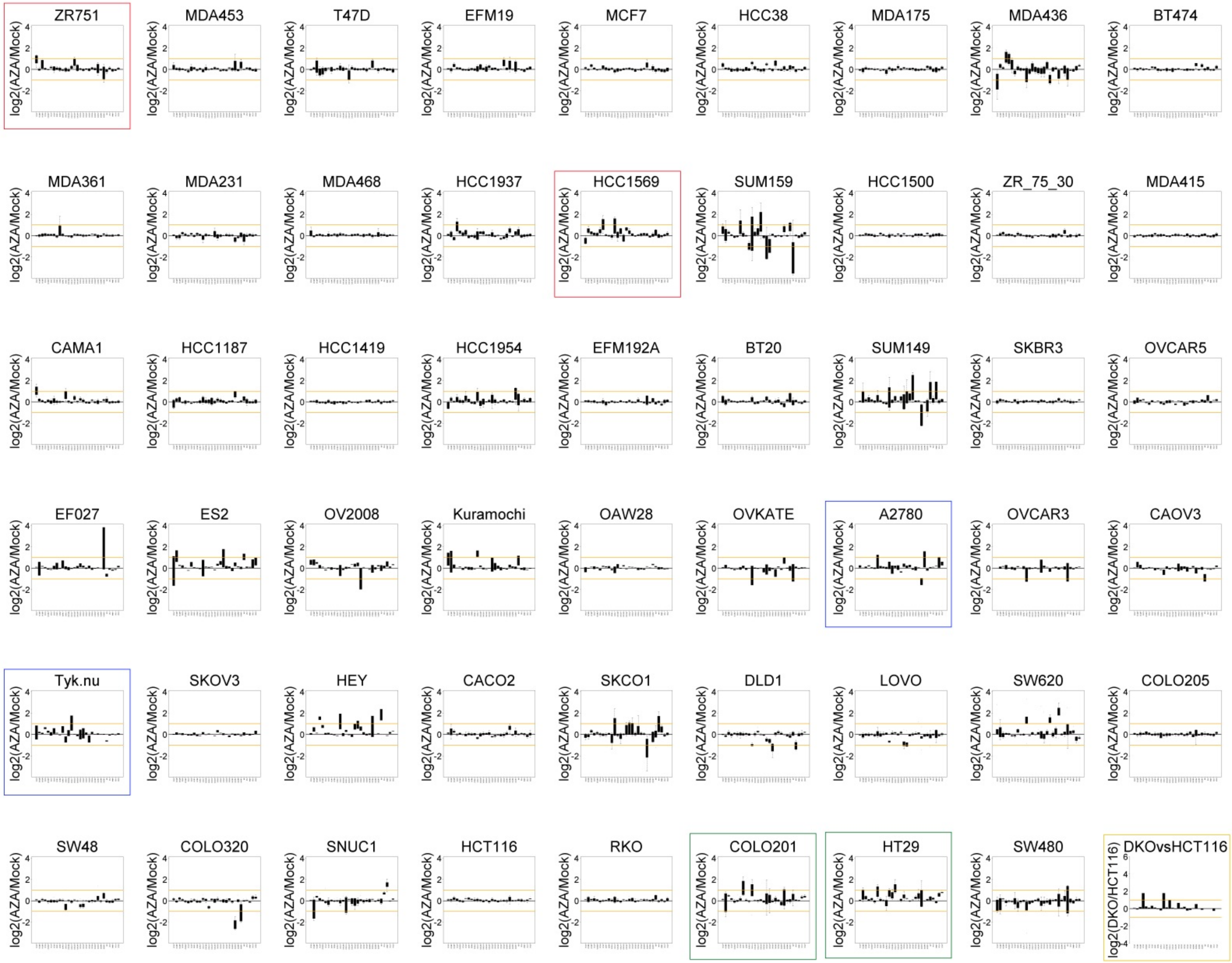

Figure S2 K

REACTOME_ANTIVIRAL_MECHANISM_BY_IFN_
STIMULATED_GENES AIM Genes

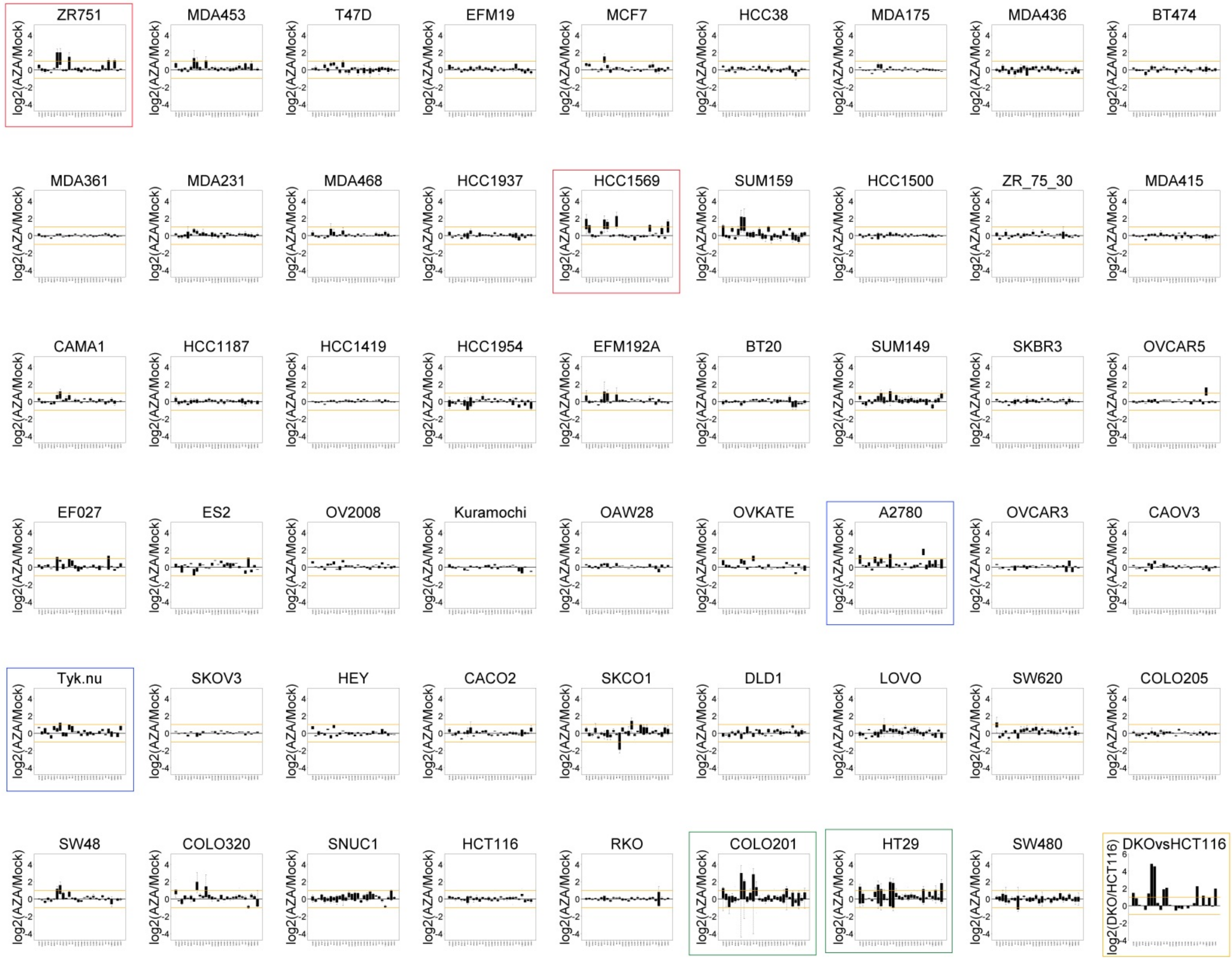

Figure S2 L

REACTOME_ANTIGEN_PROCESSING_CROSS_PRESENTATION AIM Genes

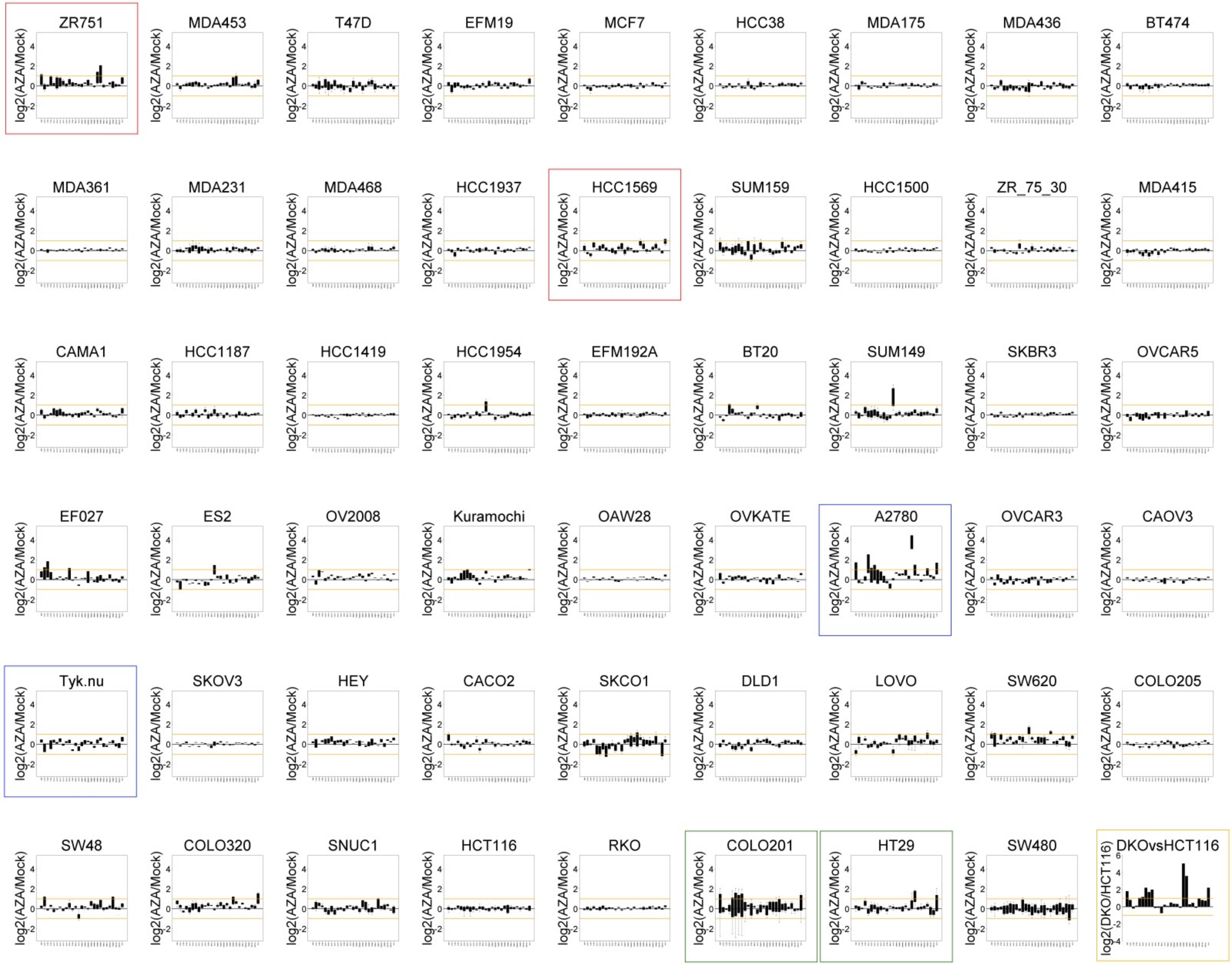

Figure S2M

KEGG_GRAFT_VERSUS_HOST_DISEASE AIM Genes

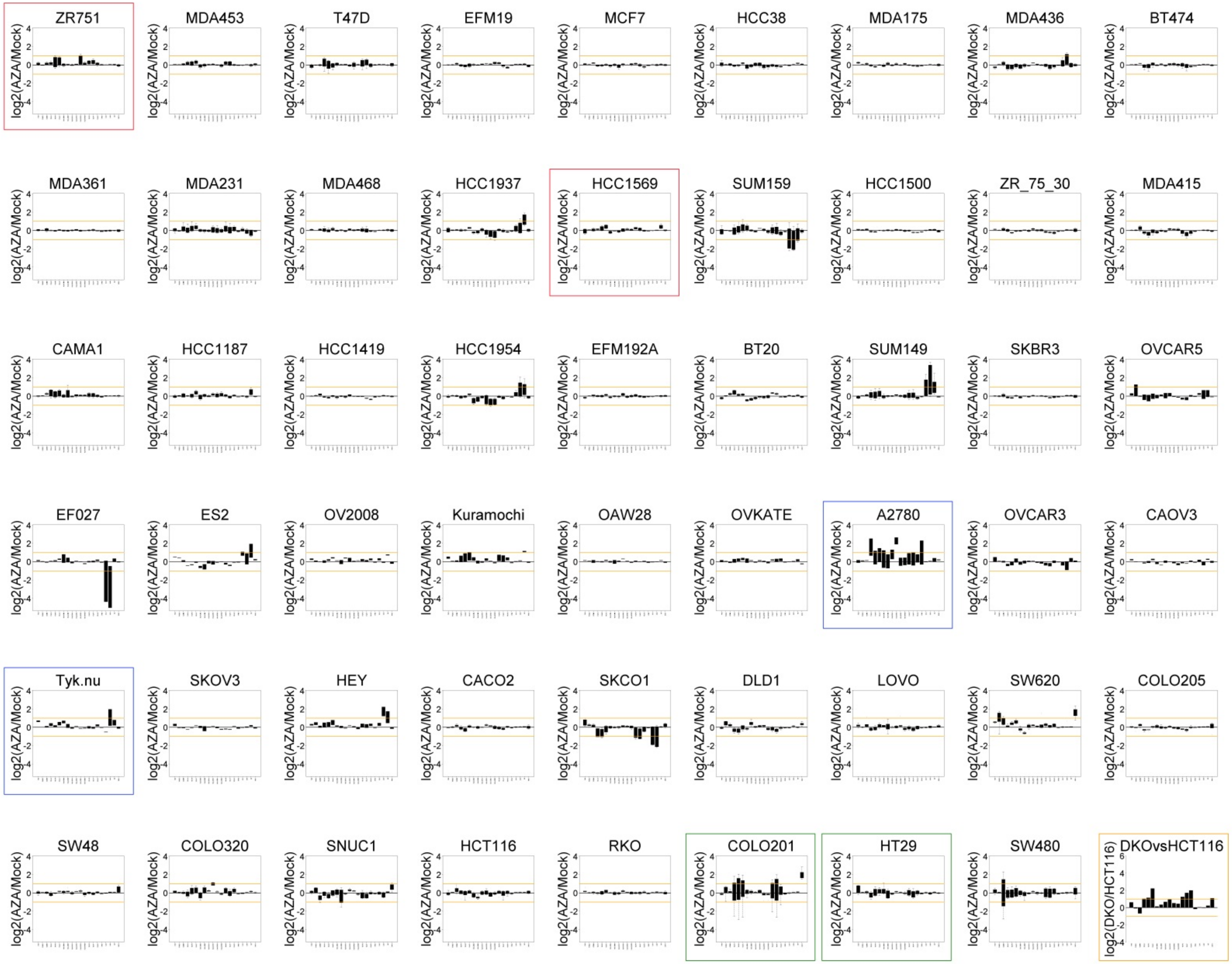

Figure S2 N

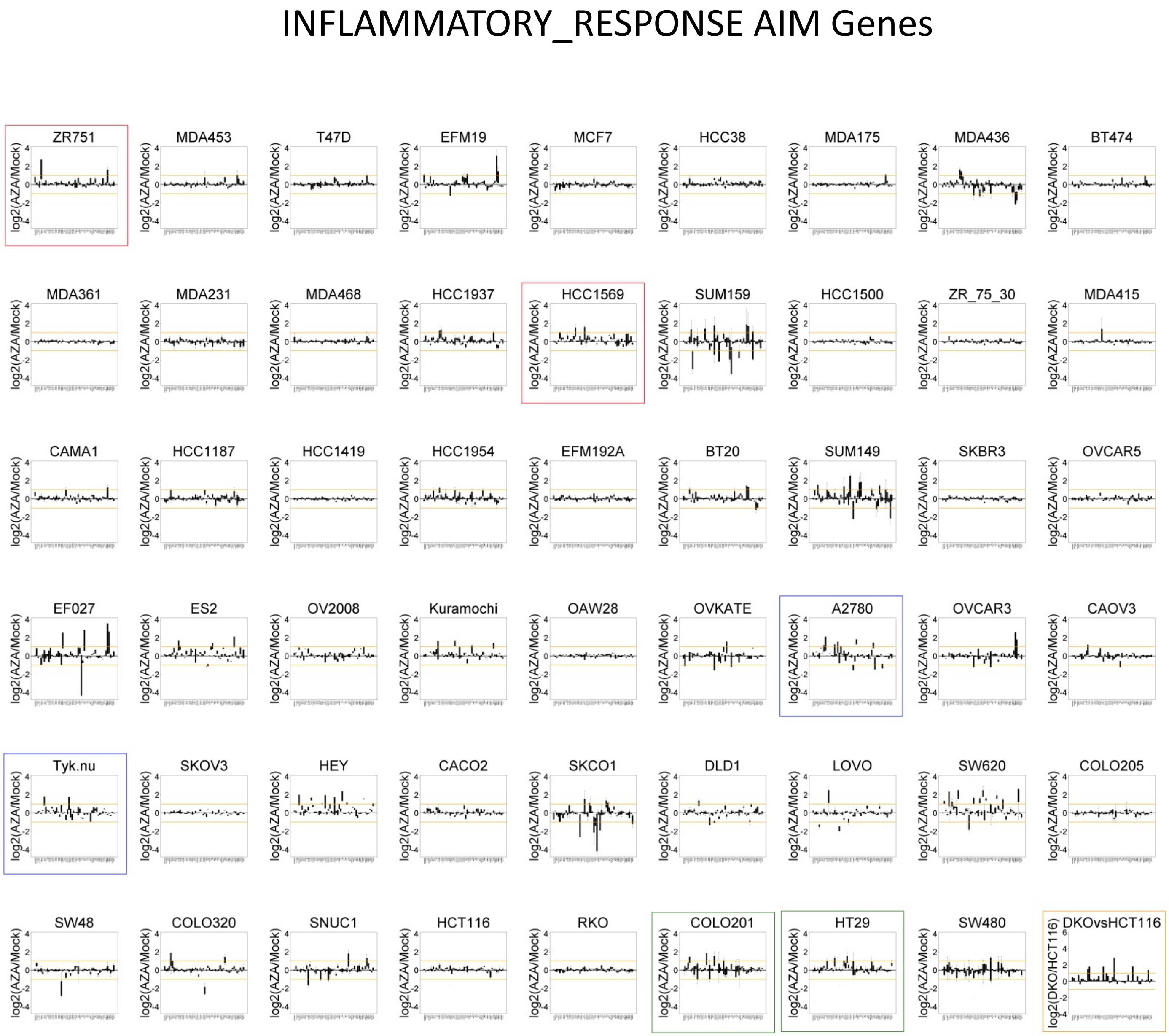
INFLAMMATORY_RESPONSE AIM Genes
ZR751
MDA453
T47D
EFM19
MCF7
HCC38
MDA175
MDA436
BT474
MDA361
MDA231
MDA468
HCC1937
HCC1569
SUM159
HCC1500
ZR_75_30
MDA415
CAMA1
HCC1187
HCC1419
HCC1954
EFM192A
BT20
SUM149
SKBR3
OVCAR5
EF027
ES2
OV2008
Kuramochi
OAW28
OVKATE
A2780
OVCAR3
CAOV3
Tyk.nu
SKOV3
HEY
CACO2
SKCO1
DLD1
LOVO
SW620
COLO205
SW48
COLO320
SNUC1
HCT116
RKO
COLO201
HT29
SW480
DKOvsHCT116
log2(AZA/Mock)
log2(DKO/HCT116)

Figure S2 O

DEFENSE_RESPONSE AIM Genes

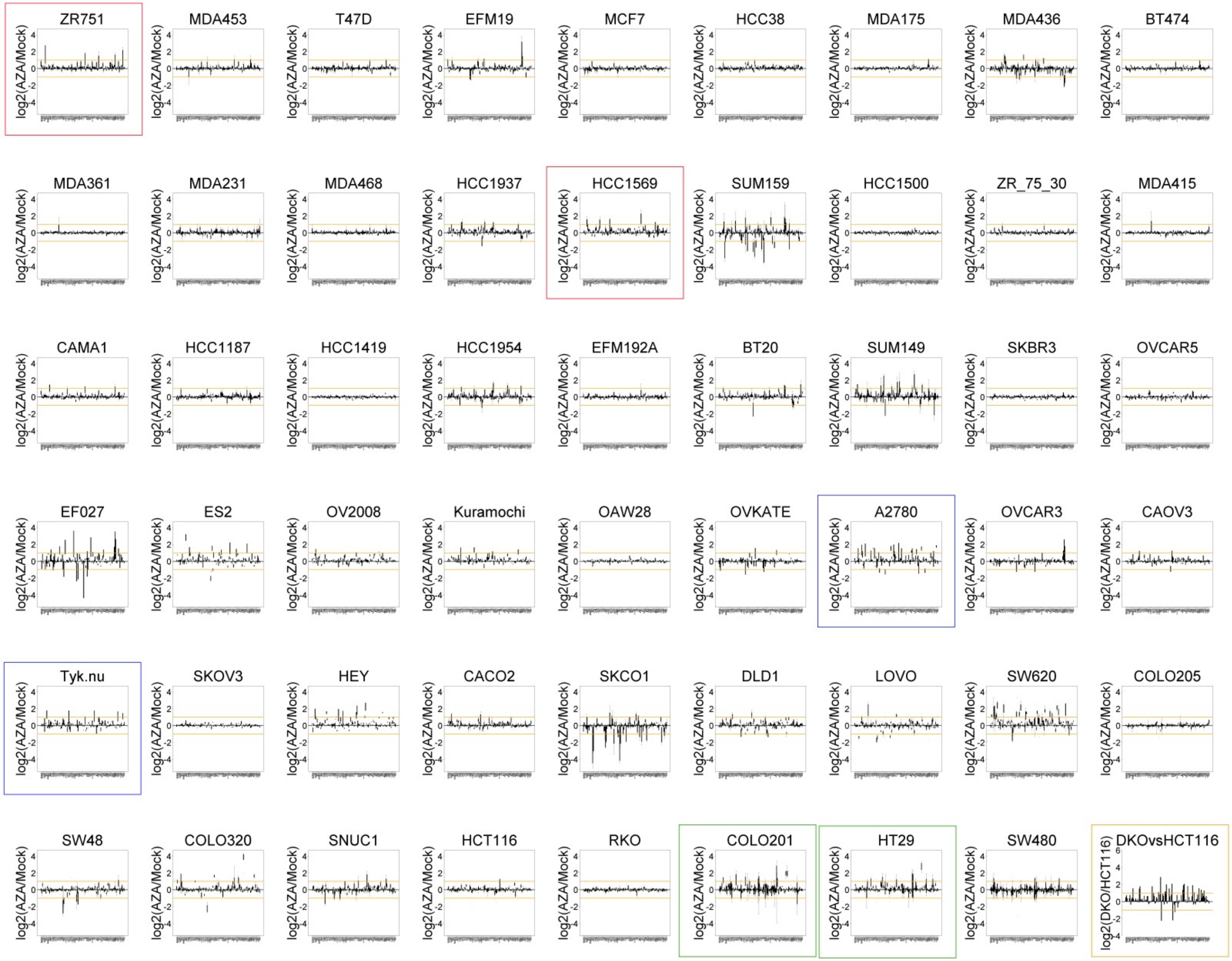

Figure S2 P

CTA Genes

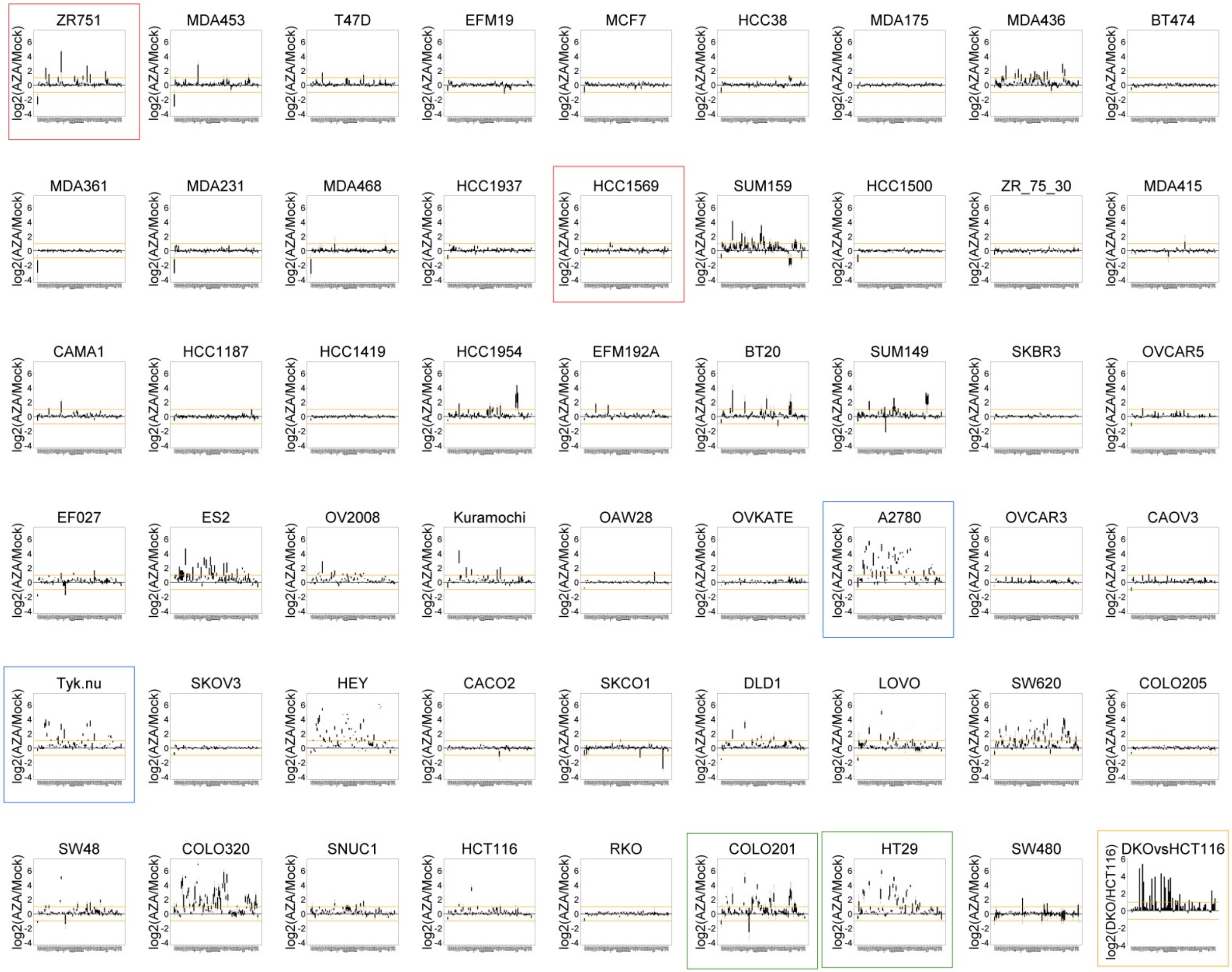

Figure S2: Plots of AZA inducible genes for each group of GSEA pathways in each cell line. The most immunogenic cell lines selected for validation/ further study are highlighted with colored boxes. ZR751 and HCC1569 breast cancer cell lines are denoted in red, A2780 and TykNu ovarian cancer cell lines in green, COLO201 and HT29 colon cancer cell lines in blue, and DKO in orange.

A

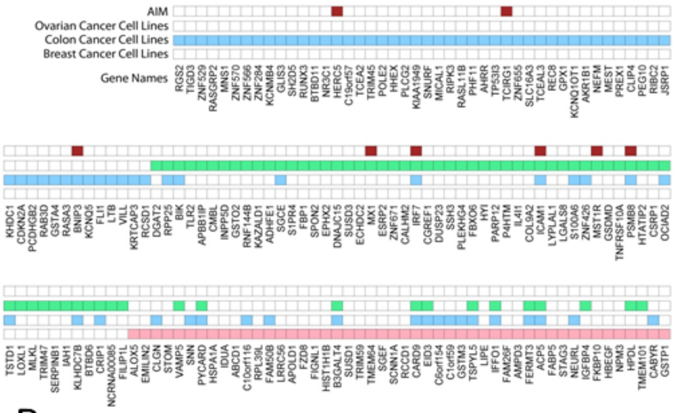

C

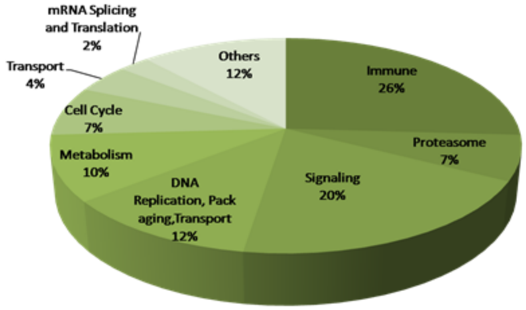

B

	demethylated & reexpressed at least 1 of the 162 genes	demethylated & reexpressed at least 1 of the 42 IMMUNE genes	Total Number of Cell Lines
Breast	9	8	26
Colorectal	7	6	14
Ovarian	10	7	13

Figure S3. A) Distribution of demethylated/reexpressed genes (162 genes) in cell lines across all three cancers. Bars indicate whether the genes are demethylated and reexpressed in breast (red), colorectal (blue), or ovarian (green) cancer cell lines after AZA treatment. Dark red bar indicates that the demethylated and reexpressed genes are also in the AIM panel. B) Number of cell lines in each tumor type that had at least one gene demethylated and reexpressed, and at least one immune gene demethylated and reexpressed. C) Gene expression categories and percentages of the demethylated/reexpressed genes.

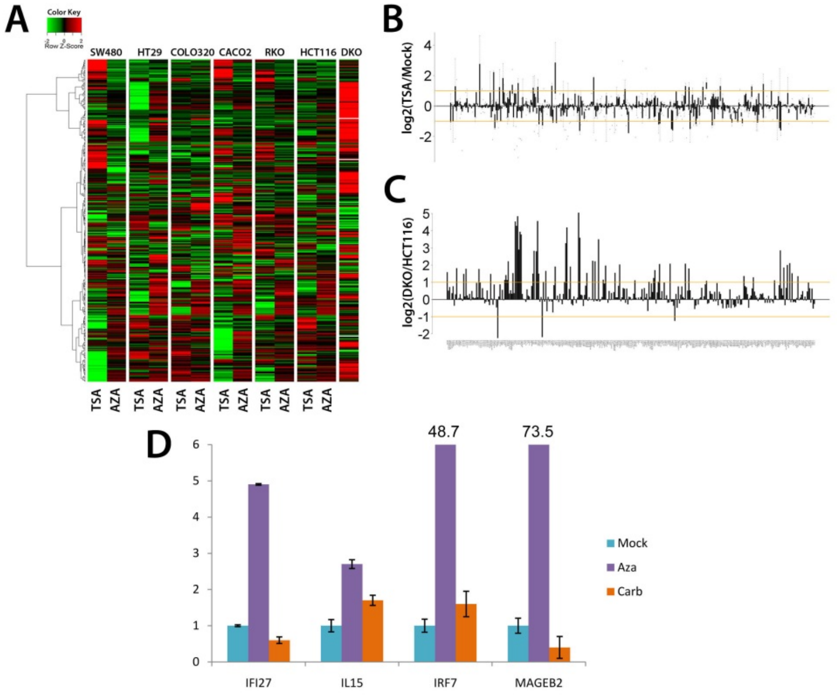

Figure S4: AIM genes are induced by TSA but not by chemotherapeutic agents. A) Six colon cancer cell lines were treated with 300 nM TSA for 18 hours; heat map shows levels of AIM genes after TSA compared to AZA treatment or the genetic mimic DKO (DNMT1+/- DNMT3-/-) cell line. B) Box plots of AIM genes after TSA treatment. C) Box plots of AIM genes in DKO cells compared to the parent HCT116 cell line. The orange lines indicate 2fold change. D) A2780 ovarian cancer cells were treated with 500 nM AZA or carboplatin for 72 hours, then allowed to recover for 7 days without drug before RNA was isolated. qRT-PCR was performed for IFI27, IL-15, IRF7, and MAGEB2 genes. Blue bars indicate Mock treatment, purple indicate AZA, and orange indicate carboplatin. Fold change is plotted on the Y axis. Each bar represents the average and standard deviation of three biological replicates.

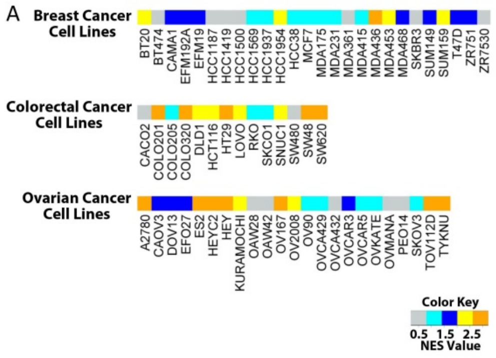

B

	Cell Line Number (NES>2.15)	Total Cell Line Number
Breast	5	26
Colorectal	9	14
Ovarian	9	23

Figure S5: GSEA analysis of cancer testis antigens shows that the pathway is upregulated (NES > 2.15, FDR < 0.25) by AZA in breast, colorectal, and ovarian cancer cell lines. The colored rectangle corresponding to NES is graded from gray (weak) to orange (strong).

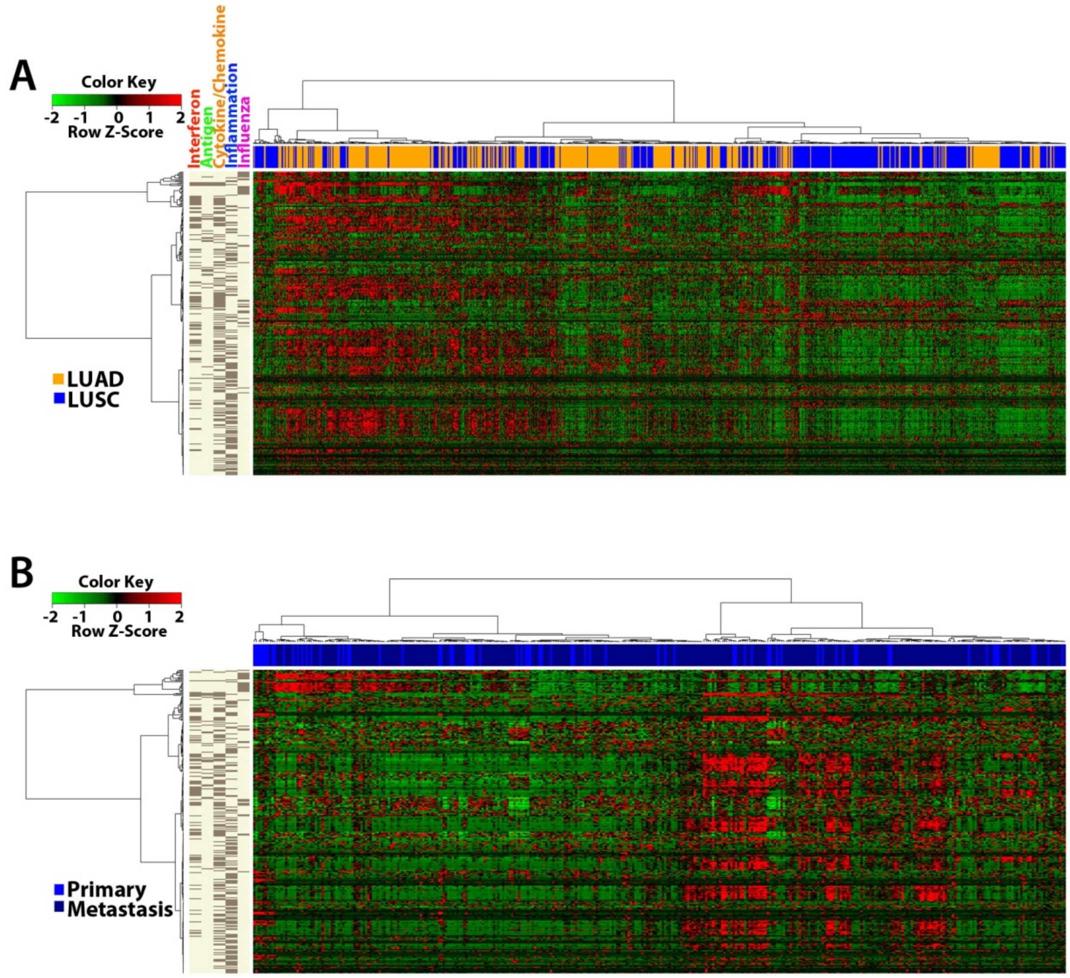

Figure S6: The AIM genes separate lung and melanoma TCGA tumors into distinct clusters. Tumors from The Cancer Genome Atlas (A) non-small cell lung cancers, B) melanomas) cluster into “high” and “low” expressing immune signatures based on AIM gene expression. The bars on the far left show the five sets of AIM genes driving the clustering. The shades of blue and orange bars at the top denote squamous versus adenocarcinoma for lung cancer, (LUAD = adenocarcinoma, LUSC = squamous) and primary tumor (light blue) versus metastasis (dark blue) for melanoma. The heat map shows transcript levels from green (low) to red (high).
